# Myalgic Encephalomyelitis/Chronic Fatigue Syndrome After SARS-CoV-2 Infection

**DOI:** 10.1001/jamanetworkopen.2024.23555

**Published:** 2024-07-24

**Authors:** Elizabeth R. Unger, Jin-Mann S. Lin, Lauren E. Wisk, Huihui Yu, Michelle L’Hommedieu, Helen Lavretsky, Juan Carlos C. Montoy, Michael A. Gottlieb, Kristin L. Rising, Nicole L. Gentile, Michelle Santangelo, Arjun K. Venkatesh, Robert M. Rodriguez, Mandy J. Hill, Rachel E. Geyer, Efrat R. Kean, Sharon Saydah, Samuel A. McDonald, Ryan Huebinger, Ahamed H. Idris, Jocelyn Dorney, Bala Hota, Erica S. Spatz, Kari A. Stephens, Robert A. Weinstein, Joann G. Elmore

**Affiliations:** 1 Division of High-Consequence Pathogens and Pathology, National Center for Emerging and Zoonotic Infectious Diseases, Centers for Disease Control and Prevention, Atlanta, Georgia; 2 Division of General Internal Medicine & Health Services Research, David Geffen School of Medicine at the University of California, Los Angeles; 3 Center for Outcomes Research & Evaluation (CORE), Section of Cardiovascular Medicine, Department of Internal Medicine, Yale School of Medicine, New Haven, Connecticut; 4 Department of Psychiatry and Biobehavioral Sciences, David Geffen School of Medicine at the University of California, Los Angeles; 5 Department of Emergency Medicine, University of California, San Francisco; 6 Department of Emergency Medicine, Rush University Medical Center, Chicago, Illinois; 7 Department of Emergency Medicine, Thomas Jefferson University, Philadelphia, Pennsylvania; 8 Jefferson Center for Connected Care, Sidney Kimmel Medical School, Thomas Jefferson University, Philadelphia, Pennsylvania; 9 Post-COVID Rehabilitation and Recovery Clinic, University of Washington, Seattle; 10 Department of Family Medicine, University of Washington, Seattle; 11 Department of Laboratory Medicine and Pathology, University of Washington, Seattle; 12 Division of Infectious Diseases, Department of Internal Medicine, Rush University Medical Center, Chicago, Illinois; 13 Department of Emergency Medicine, Yale School of Medicine, New Haven, Connecticut; 14 Department of Emergency Medicine, UTHealth Houston, Houston, Texas; 15 National Center for Immunizations and Respiratory Diseases, Centers for Disease Control and Prevention, Atlanta, Georgia; 16 Department of Emergency Medicine, University of Texas Southwestern Medical Center, Dallas; 17 Clinical Informatics Center, University of Texas Southwestern Medical Center, Dallas; 18 Tendo Systems, Inc, Philadelphia, Pennsylvania; 19 Department of Epidemiology, Yale School of Public Health, New Haven, Connecticut; 20 Department of Biomedical Informatics and Medical Education, University of Washington, Seattle; 21 Division of Infectious Diseases, Department of Medicine, Cook County Hospital, Chicago, Illinois

## Abstract

**Importance:**

Chronic symptoms reported following an infection with SARS-CoV-2, such as cognitive problems, overlap with symptoms included in the definition of myalgic encephalomyelitis/chronic fatigue syndrome (ME/CFS).

**Objective:**

To evaluate the prevalence of ME/CFS-like illness subsequent to acute SARS-CoV-2 infection, changes in ME/CFS symptoms through 12 months of follow-up, and the association of ME/CFS symptoms with SARS-CoV-2 test results at the acute infection–like index illness.

**Design, Setting, and Participants:**

This prospective, multisite, longitudinal cohort study (Innovative Support for Patients with SARS-CoV-2 Infections Registry [INSPIRE]) enrolled participants from December 11, 2020, to August 29, 2022. Participants were adults aged 18 to 64 years with acute symptoms suggestive of SARS-CoV-2 infection who received a US Food and Drug Administration–approved SARS-CoV-2 test at the time of illness and did not die or withdraw from the study by 3 months. Follow-up surveys were collected through February 28, 2023.

**Exposure:**

COVID-19 status (positive vs negative) at enrollment.

**Main Outcome and Measures:**

The main outcome was the weighted proportion of participants with ME/CFS-like illness based on the 2015 Institute of Medicine clinical case definition using self-reported symptoms.

**Results:**

A total of 4378 participants were included in the study. Most were female (3226 [68.1%]). Mean (SD) age was 37.8 (11.8) years. The survey completion rates ranged from 38.7% (3613 of 4738 participants) to 76.3% (1835 of 4738) and decreased over time. The weighted proportion of participants identified with ME/CFS-like illness did not change significantly at 3 through 12 months of follow-up and was similar in the COVID-19–positive (range, 2.8%-3.7%) and COVID-19–negative (range, 3.1%-4.5%) groups. Adjusted analyses revealed no significant difference in the odds of ME/CFS-like illness at any time point between COVID-19–positive and COVID-19–negative individuals (marginal odds ratio range, 0.84 [95% CI, 0.42-1.67] to 1.18 [95% CI, 0.55-2.51]).

**Conclusions and Relevance:**

In this prospective cohort study, there was no evidence that the proportion of participants with ME/CFS-like illness differed between those infected with SARS-CoV-2 vs those without SARS-CoV-2 infection up to 12 months after infection. A 3% to 4% prevalence of ME/CFS-like illness after an acute infection–like index illness would impose a high societal burden given the millions of persons infected with SARS-CoV-2.

## Key Points

**Question:**

Does prevalence of myalgic encephalomyelitis/chronic fatigue syndrome (ME/CFS)–like illness differ between individuals with an acute infection–like index illness who are COVID-19 positive or negative?

**Findings:**

In this cohort study of 4378 participants, the weighted prevalence of ME/CFS-like illness was 4.5% or less at 3 to 12 months after the index illness in the COVID-19–positive and COVID-19–negative groups, with no significant differences in odds of ME/CFS-like illness.

**Meaning:**

The findings suggest that ME/CFS-like illness following an acute infection–like index illness does not vary by COVID-19 test result.

## Teaser

This cohort study investigates the incidence of myalgic encephalomyelitis/chronic fatigue syndrome (ME/CFS)–like symptoms in individuals tested for SARS-CoV-2 infection and whether ME/CFS symptom prevalence is associated with positive or negative COVID-19 test results.

## Introduction

Chronic medical syndromes can occur after a variety of acute infections (eg, postpolio syndrome, post–Epstein Barr virus syndrome).^1,2^ These postacute infection syndromes (PAISs) share similar symptoms, including functional impairment associated with fatigue, exertion intolerance, and cognitive problems. Myalgic encephalomyelitis/chronic fatigue syndrome (ME/CFS), a complex, chronic, debilitating condition with systemic manifestations often linked to a prior acute influenza-like illness,^3,4^ is emblematic of the largely enigmatic group of PAISs. Fatigue is the most common symptom among patients with post-COVID condition or long COVID,^5,6,7^ and the symptom profile of postacute sequelae of COVID-19 and of other PAISs overlaps with characteristic symptoms of ME/CFS. The prevalence of long COVID symptoms in the US,^8^ including those overlapping with ME/CFS symptoms, suggests that millions of individuals will be impacted, with medical costs in the billions,^9^ emphasizing the need to understand PAISs.

The COVID-19 pandemic raised awareness of PAISs and provides a unique opportunity to examine the occurrence of ME/CFS following a specific infection. Through the Innovative Support for Patients with SARS-CoV-2 Infections Registry (INSPIRE) study, we collected thousands of self-reported outcome data points, allowing the identification and evaluation of patients with ME/CFS-like illness. The objective of this analysis was to evaluate the occurrence of ME/CFS among INSPIRE participants following symptomatic acute illness that prompted their COVID-19 test and to compare the odds of ME/CFS in the COVID-19–positive cohort and the COVID-19–negative cohort.

## Methods

### Study Design and Data Source

INSPIRE was a multicenter, prospective, longitudinal registry cohort study that enrolled individuals who experienced an acute index illness suggestive of COVID-19 between December 11, 2020, and August 29, 2022. Eight geographically diverse study sites across the US recruited and enrolled participants with a protocol and methods previously described.^10^ Recruitment occurred in person, by email or telephone, and through electronic advertisement. A secure online platform (Hugo; Hugo Health LLC) facilitated collection of consent and survey distribution. Participants self-enrolled by first completing an online eligibility screener, during which they were asked to self-report COVID-19–like symptoms that they experienced within the past 42 days and provide documentation of their valid COVID-19 test and test result. If respondents were deemed eligible for participation, they were provided a consent form on the online platform. Follow-up surveys were collected through February 28, 2023. The institutional review board at each of the study sites reviewed and approved this study. The Centers for Disease Control and Prevention (CDC) reviewed the project and determined the study was nonengaged human participants research. The study followed the Strengthening the Reporting of Observational Studies in Epidemiology (STROBE) reporting guideline for cohort studies.^11^

### Cohort Definition

The INSPIRE study included adult participants (aged ≥18 years) who were fluent in English or Spanish, had access to an internet-enabled device to allow for participation, and had self-reported symptoms suggestive of acute SARS-CoV-2 infection at the time of their SARS-CoV-2 test. Participants had to have been tested for SARS-CoV-2 with a US Food and Drug Administration–approved or authorized molecular or antigen-based assay within 42 days before their study enrollment. For this analysis, we excluded participants aged 65 years or older to minimize the confounding impact of age-associated illnesses that could explain ME/CFS symptoms.^12,13,14^ We excluded participants who did not link their electronic health portal connection with the online platform, did not complete the baseline survey, died or withdrew from the study before 3 months, or did not have valid COVID-19 test results. The flowchart (eFigure 1 in Supplement 1) has detailed information on recruitment and follow-up completion.

Participants were grouped as either COVID-19 positive or COVID-19 negative based on their index SARS-CoV-2 test result. If they had more than 1 test within 7 days of enrollment and results were discordant, we considered the positive test result to be the true result. If a participant’s test result changed more than 7 days after enrollment, we kept the participant in their initial group and adjusted for subsequent positive test results as a covariate.

### Variables

Participants completed surveys at baseline and at 4 quarterly follow-up times. Participants self-reported sociodemographic data, including age, gender (female, male, or transgender, nonbinary, or other gender), race (Asian, Native Hawaiian, or Other Pacific Islander; Black or African American; White; or other [groups are listed in eAppendix 3 in Supplement 1] or multiracial), ethnicity (Hispanic, Latino, or of Spanish origin or not Hispanic, Latino, or of Spanish origin), educational level, income, employment status, health insurance status, and marital status. Self-reported race and ethnicity items on the baseline survey were included because patient outcomes have been reported to vary across racial and ethnic groups. Standardized questions assessing physical and mental health, symptoms, access to care, and work-related outcomes were included in the baseline and follow-up surveys.^15^ Participants listed other conditions that could contribute to ME/CFS symptoms in free-text responses (a list of the free-text entries is included in eAppendix 2 in Supplement 1). The Patient-Reported Outcomes Measurement Information System–29 (PROMIS-29) profile, version 2.1 was administered at baseline and at all follow-up times.^16,17^ It contains 7 domains, including symptom-oriented (sleep, pain, anxiety, depression, and energy or fatigue) and function-oriented (physical function and social role or activity limitation) measures. We assessed cognitive function via the PROMIS Short Form–Cognitive Function 8a measure. The PROMIS measures use T-score metrics with a mean of 50 and SD of 10 in a reference population (ie, the US general population). Higher scores indicate a greater degree of the concept being measured (eg, better functioning for the physical function subscale, more depressed for the depression subscale).

### ME/CFS Outcomes of Interest

We used the 2015 Institute of Medicine (IOM) criteria^15^ for binary classification of the primary ME/CFS outcome, operationalized using participants’ responses to symptoms from the CDC ME/CFS Symptom Screener–Short Form, version 1.2 (eAppendix 1 in Supplement 1) and the Physical Function subscale of the PROMIS-29, version 2.1^18^ profile (algorithm in eTable 1 in Supplement 1). In brief, the criteria include activity limitations associated with fatigue, postexertional malaise, and sleep problems as well as either cognitive impairment or orthostatic intolerance. Because ME/CFS diagnosis requires a full clinical evaluation to identify treatable conditions contributing to symptoms used in diagnosis, the self-reported information in this study only allowed determination of ME/CFS-like illness, hereafter referred to as *ME/CFS*. Additionally, the survey questions did not allow clear detection of the presence of chronic symptoms before participants’ acute index illness. The count of ME/CFS diagnostic criteria met (0-5) was also used as an outcome for this study.

For simplicity of monitoring ME/CFS-related symptoms, we grouped participants as ever having ME/CFS symptoms if they met criteria at any time point (acute index illness through 12 months) or never having ME/CFS symptoms if they did not meet criteria at any time point. We focused regression analysis results comparing ME/CFS classification at 3 months and beyond but, for parsimony, included those who ever met the ME/CFS definition at any time point to summarize participant characteristics descriptively.

### Statistical Analysis

Statistical analyses were conducted using SAS, version 9.4 (SAS Institute Inc). All tests were 2-sided with a significance threshold of *P* = .05. Bivariate analyses were performed to examine the association between participant characteristics and ME/CFS status at any time point and whether characteristics were balanced between COVID-19–positive and COVID-19–negative participants. This identified significant differences between the COVID-19–positive and COVID-19–negative groups in baseline characteristics that were associated with ME/CFS outcomes. To address the imbalanced distribution of these confounders, we used the inverse propensity score weighting (IPW) technique. We assessed (Table 1 and eTable 2 and eFigure 2 in Supplement 1) how well the distribution of confounders and covariates were balanced between the COVID-19–positive and COVID-19–negative groups through IPW. Because of the need for balancing groups by COVID-19 status using IPW, all results in the main text are weighted, and observed (unweighted) results are included in eTable2 in Supplement 1.

**Table 1.** Weighted Distribution of Confounders and Covariates Between COVID-19 Groups^a^

Characteristic	Participants, No. (%)			
	Overall (N = 4738)	COVID-19 positive (n = 2418)	COVID-19 negative (n = 2320)	*P* value
Age at enrollment, y				
18-34	2080 (43.9)	1088 (45.0)	992 (42.8)	.27
35-49	1703 (36.0)	846 (35.0)	858 (37.0)	
50-64	955 (20.2)	485 (20.1)	470 (20.2)	
Gender				
Female	3236 (68.3)	1654 (68.4)	1582 (68.2)	.99
Male	1435 (30.3)	730 (30.2)	705 (30.4)	
Transgender, nonbinary, or other^b^	67 (1.4)	34 (1.4)	33 (1.4)	
Ethnicity				
Hispanic, Latino, or of Spanish origin	690 (14.6)	353 (14.6)	337 (14.5)	.97
Not Hispanic, Latino, or of Spanish origin	4048 (85.4)	2065 (85.4)	1983 (85.5)	
Race				
Asian, Native Hawaiian, or Other Pacific Islander	607 (12.8)	310 (12.8)	297 (12.8)	.04
Black or African American	571 (12.1)	271 (11.2)	301 (13.0)	
White	3125 (66.0)	1592 (65.9)	1533 (66.1)	
Other race or multiracial^c^	435 (9.2)	245 (10.1)	190 (8.2)	
Educational attainment				
Less than high school diploma	72 (1.5)	35 (1.4)	38 (1.6)	.71
High school graduate or GED	411 (8.7)	213 (8.8)	198 (8.5)	
Some college but did not complete degree	710 (15.0)	356 (14.7)	354 (15.3)	
2-y College degree	353 (7.5)	174 (7.2)	179 (7.7)	
4-y College degree	1485 (31.3)	781 (32.3)	703 (30.3)	
More than 4-y college degree	1707 (36.0)	859 (35.5)	848 (36.5)	
Marital status				
Never married	1832 (38.7)	938 (38.8)	894 (38.5)	.94
Married or living with a partner	2427 (51.2)	1233 (51.0)	1194 (51.5)	
Divorced, widowed, or separated	479 (10.1)	247 (10.2)	232 (10.0)	
Prepandemic family income, $				
<10 000	378 (8.0)	185 (7.7)	193 (8.3)	.42
10 000 to <35 000	580 (12.2)	294 (12.2)	286 (12.3)	
35 000 to <50 000	562 (11.9)	283 (11.7)	280 (12.0)	
50 000 to <75 000	701 (14.8)	341 (14.1)	360 (15.5)	
≥75 000	2516 (53.1)	1315 (54.4)	1201 (51.8)	
Location of COVID-19 test				
At-home testing kit	633 (13.4)	325 (13.4)	308 (13.3)	.85
Tent or drive-up testing site	2349 (49.6)	1204 (49.8)	1145 (49.3)	
Clinic, including an urgent care clinic	706 (14.9)	345 (14.3)	361 (15.6)	
Hospital	385 (8.1)	199 (8.2)	186 (8.0)	
Emergency department	288 (6.1)	153 (6.3)	135 (5.8)	
Other	378 (8.0)	193 (8.0)	185 (8.0)	
Tobacco use in past 12 mo				
Daily or near daily	347 (7.3)	173 (7.1)	174 (7.5)	.92
Weekly	96 (2.0)	47 (1.9)	49 (2.1)	
Monthly	251 (5.3)	128 (5.3)	123 (5.3)	
Less than monthly	94 (2.0)	45 (1.8)	49 (2.1)	
Not at all	3950 (83.4)	2026 (83.8)	1924 (82.9)	
Binge drinking in past 12 mo				
Daily or near daily	54 (1.1)	32 (1.3)	22 (0.9)	.23
Weekly	459 (9.7)	243 (10.1)	216 (9.3)	
Monthly	1112 (23.5)	569 (23.5)	542 (23.4)	
Less than monthly	619 (13.1)	332 (13.7)	286 (12.3)	
Not at all	2495 (52.7)	1241 (51.3)	1254 (54.0)	
Health insurance				
Private and public	83 (1.8)	38 (1.6)	45 (2.0)	.74
Private only	3603 (76.1)	1840 (76.1)	1763 (76.0)	
Public only	862 (18.2)	445 (18.4)	417 (18.0)	
None	190 (4.0)	95 (3.9)	95 (4.1)	
Hospitalized for index illness^d^				
No	3468 (73.2)	1772 (73.3)	1696 (73.1)	<.001
Yes	91 (1.9)	64 (2.6)	27 (1.1)	
Missing	1180 (24.9)	582 (24.1)	598 (25.8)	
Variant period at index test date^e^				
Pre-Delta	827 (17.4)	430 (17.8)	396 (17.1)	.57
Delta	1577 (33.3)	814 (33.7)	762 (32.9)	
Omicron	2335 (49.3)	1173 (48.5)	1161 (50.1)	
Self-reported comorbidities				
Overweight or obesity	1003 (21.2)	498 (20.6)	505 (21.8)	.33
Asthma, moderate or severe	447 (9.4)	234 (9.7)	213 (9.2)	.55
Hypertension or high blood pressure	443 (9.3)	215 (8.9)	228 (9.8)	.26
Current tobacco use^f^	187 (4.0)	91 (3.8)	96 (4.2)	.48
Diabetes	183 (3.9)	83 (3.4)	100 (4.3)	.13
Heart conditions^g^	65 (1.4)	34 (1.4)	31 (1.3)	.90
Kidney disease	47 (1.0)	24 (1.0)	24 (1.0)	.90
Liver disease	25 (0.5)	11 (0.5)	13 (0.6)	.60
Emphysema or COPD	23 (0.5)	12 (0.5)	11 (0.5)	.87
Other conditions^h^	361 (7.6)	174 (7.2)	187 (8.0)	.28
Symptoms before index illness				
Fatigue, tiredness, or exhaustion	1115 (23.5)	561 (23.2)	554 (23.9)	.58
Problems getting to sleep	1050 (22.2)	556 (23.0)	494 (21.3)	.16
Unrefreshing sleep	740 (15.6)	383 (15.8)	357 (15.4)	.68
Muscle aches or muscle pains	545 (11.5)	272 (11.2)	273 (11.8)	.58
Pain in joints	361 (7.6)	178 (7.4)	183 (7.9)	.49
Difficulty thinking or concentrating	308 (6.5)	150 (6.2)	158 (6.8)	.41
Forgetfulness or memory problems	246 (5.2)	125 (5.2)	121 (5.2)	.94
Dizziness or fainting	244 (5.1)	121 (5.0)	123 (5.3)	.68

Abbreviations: COPD, chronic obstructive pulmonary disease; GED, General Educational Development.

^a^ Weighted by inverse propensity scores calculated by accounting for the confounders and covariates in the table. Variables correlated with COVID-19 status but not with the myalgic encephalomyelitis/chronic fatigue syndrome outcomes were excluded from propensity score calculation to avoid introducing noise into estimating the impact of COVID-19 infection and therefore are not included in this table. Hospitalization for the index illness was not well balanced by inverse propensity score weighting and was therefore included in the generalized estimating equation modeling.

^b^ Other gender included gender nonconforming, not listed, or preferred not to answer.

^c^ Other races listed in free-text responses entered by participants are included in eAppendix 3 in Supplement 1.

^d^ Hospitalization for the index illness was a new question added to the 3-month survey after April 14, 2021. There were 1134 participants missing a response to this question that were included as a separate category in the analysis.

^e^ Viral prevalence greater than 50% was used to determine the dominant variant.

^f^ Any type of tobacco, including smokeless tobacco.

^g^ Included coronary artery disease, heart failure, and cardiomyopathy.

^h^ Other conditions listed in free-text responses entered by participants that could contribute to myalgic encephalomyelitis/chronic fatigue syndrome symptoms. A list of the free-text entries is included in eAppendix 2 in Supplement 1.

Incorporating IPW, we used generalized estimating equation (GEE) models to examine the association between initial SARS-CoV-2 infection and ME/CFS outcomes across time and how the risk of ME/CFS outcomes changed over time in each COVID-19 group after the index illness. Specifically, we used a GEE with a binomial distribution and logit link function for the binary outcome of ME/CFS and a GEE negative binomial model with the log link function for the counts of ME/CFS diagnostic criteria met (0-5). Both models included the initial SARS-CoV-2 infection status at enrollment, time points (baseline and 4 quarterly follow-up times), any postbaseline SARS-CoV-2 infection as a time-varying covariate, and hospitalization status for the index illness, which was not fully balanced by IPW (eFigure 2 in Supplement 1). Additionally, we included adjustment for the SARS-CoV-2 variant period at enrollment using previously published methods.^19^ We also allowed initial COVID-19 status to interact with time points, subsequent SARS-CoV-2 infection, and the variant period. Marginal differences between the COVID-19–positive and COVID-19–negative groups were estimated at each time point. Simultaneously, changes over time from 3 through 12 months in each COVID-19 group were estimated for all possible paired time comparisons. To examine symptom and function measures between participants with and without ME/CFS, effect sizes (ie, Cohen *d*) were calculated for group mean differences.

## Results

There were 4738 participants included in the study, of whom 3226 (68.1%) identified as female, 1437 (30.3%) as male, and 75 (1.6%) as transgender, nonbinary, or other gender. A total of 691 (14.6%) identified as Hispanic, Latino, or of Spanish origin and 4047 (85.4%) as not Hispanic, Latino, or of Spanish origin; 631 (13.3%) were Asian, Native Hawaiian, or Other Pacific Islander; 513 (10.8%), Black or African American; 3133 (66.1%), White; and 461 (9.7%), other race or multiracial. Mean (SD) age was 37.8 (11.8) years. A total of 2440 (51.5%) were married or living with a partner, and 3636 (76.7%) were privately insured (Table 2). Table 2 shows the participants’ observed baseline characteristics by ME/CFS status (ever, 322 [6.8%]; never, 4416 [93.2%]), most of which were significantly different. After IPW, the frequency of symptoms of ME/CFS reported by participants ranged from 5.1% for dizziness or fainting and 5.2% for forgetfulness or memory problems to 23.5% for fatigue (Table 1). Among all 4738 participants, the survey completion rates ranged from 38.7% (3613) to 76.3% (1835) and decreased over time. Less than one-third (1357 [28.6%]) provided data at all follow-up time points

**Table 2.** Observed Characteristics by Ever or Never Meeting Criteria for ME/CFS

Characteristic	Participants, No. (%)			*P* value
	Overall (N = 4738)	ME/CFS classification^a^		
		Ever (n = 322)	Never (n = 4416)	
Age at enrollment, y				
18-34	2161 (45.6)	110 (34.2)	2051 (46.4)	<.001
35-49	1647 (34.8)	124 (38.5)	1523 (34.5)	
50-64	930 (19.6)	88 (27.3)	842 (19.1)	
Gender				
Female	3226 (68.1)	266 (82.6)	2960 (67.0)	<.001
Male	1437 (30.3)	49 (15.2)	1388 (31.4)	
Transgender, nonbinary, or other^b^	75 (1.6)	7 (2.2)	68 (1.5)	
Ethnicity				
Hispanic, Latino, or of Spanish origin	691 (14.6)	66 (20.5)	625 (14.2)	.002
Not Hispanic, Latino, or of Spanish origin	4047 (85.4)	256 (79.5)	3791 (85.8)	
Race				
Asian, Native Hawaiian, or Other Pacific Islander	631 (13.3)	18 (5.6)	613 (13.9)	<.001
Black or African American	513 (10.8)	36 (11.2)	477 (10.8)	
White	3133 (66.1)	222 (68.9)	2911 (65.9)	
Other race or multiracial^c^	461 (9.7)	46 (14.3)	415 (9.4)	
Educational attainment				
Less than high school diploma	65 (1.4)	6 (1.9)	59 (1.3)	<.001
High school graduate or GED	404 (8.5)	37 (11.5)	367 (8.3)	
Some college but did not complete degree	663 (14.0)	73 (22.7)	590 (13.4)	
2-y College degree	350 (7.4)	45 (14.0)	305 (6.9)	
4-y College degree	1553 (32.8)	77 (23.9)	1476 (33.4)	
More than 4-y college degree	1703 (35.9)	84 (26.1)	1619 (36.7)	
Marital status				
Never married	1858 (39.2)	110 (34.2)	1748 (39.6)	<.001
Married or living with a partner	2440 (51.5)	154 (47.8)	2286 (51.8)	
Divorced, widowed, or separated	440 (9.3)	58 (18.0)	382 (8.7)	
Prepandemic family income, $				
<10 000	348 (7.3)	31 (9.6)	317 (7.2)	<.001
10 000 to <35 000	580 (12.2)	72 (22.4)	508 (11.5)	
35 000 to <50 000	540 (11.4)	52 (16.1)	488 (11.1)	
50 000 to <75 000	658 (13.9)	46 (14.3)	612 (13.9)	
≥75 000	2612 (55.1)	121 (37.6)	2491 (56.4)	
Location of COVID-19 test				
At-home testing kit	638 (13.5)	29 (9.0)	609 (13.8)	<.001
Tent or drive-up testing site	2380 (50.2)	132 (41.0)	2248 (50.9)	
Clinic, including an urgent care clinic	656 (13.8)	59 (18.3)	597 (13.5)	
Hospital	402 (8.5)	33 (10.2)	369 (8.4)	
Emergency department	267 (5.6)	45 (14.0)	222 (5.0)	
Other	395 (8.3)	24 (7.5)	371 (8.4)	
Tobacco use in past 12 mo				
Daily or near daily	321 (6.8)	45 (14.0)	276 (6.3)	<.001
Weekly	94 (2.0)	10 (3.1)	84 (1.9)	
Monthly	253 (5.3)	15 (4.7)	238 (5.4)	
Less than monthly	81 (1.7)	9 (2.8)	72 (1.6)	
Not at all	3989 (84.2)	243 (75.5)	3746 (84.8)	
Binge drinking in past 12 mo				
Daily or near daily	65 (1.4)	4 (1.2)	61 (1.4)	<.001
Weekly	487 (10.3)	27 (8.4)	460 (10.4)	
Monthly	1109 (23.4)	52 (16.1)	1057 (23.9)	
Less than monthly	666 (14.1)	24 (7.5)	642 (14.5)	
Not at all	2411 (50.9)	215 (66.8)	2196 (49.7)	
Health insurance				
Private and public	74 (1.6)	13 (4.0)	61 (1.4)	<.001
Private only	3636 (76.7)	165 (51.2)	3471 (78.6)	
Public only	835 (17.6)	123 (38.2)	712 (16.1)	
None	193 (4.1)	21 (6.5)	172 (3.9)	
Hospitalized during index illness^d^				
No	3478 (73.4)	226 (70.2)	3252 (73.6)	<.001
Yes	126 (2.7)	29 (9.0)	97 (2.2)	
Missing	1134 (23.9)	67 (20.8)	1067 (24.2)	
Variant period at index test date^e^				
Pre-Delta	818 (17.3)	76 (23.6)	742 (16.8)	.005
Delta	1613 (34.0)	109 (33.9)	1504 (34.1)	
Omicron	2307 (48.7)	137 (42.5)	2170 (49.1)	
Self-reported comorbidities				
Overweight or obesity	967 (20.4)	112 (34.8)	855 (19.4)	<.001
Asthma, moderate or severe	466 (9.8)	74 (23.0)	392 (8.9)	<.001
Hypertension or high blood pressure	421 (8.9)	50 (15.5)	371 (8.4)	<.001
Current tobacco use^f^	169 (3.6)	29 (9.0)	140 (3.2)	<.001
Diabetes	168 (3.5)	21 (6.5)	147 (3.3)	.003
Heart conditions^g^	69 (1.5)	10 (3.1)	59 (1.3)	.01
Kidney disease	40 (0.8)	5 (1.6)	35 (0.8)	.15
Liver disease	30 (0.6)	4 (1.2)	26 (0.6)	.15
Emphysema or COPD	18 (0.4)	7 (2.2)	11 (0.2)	<.001
Other conditions in free text^h^	336 (7.1)	44 (13.7)	292 (6.6)	<.001
Symptoms before index COVID-19 test				
Fatigue, tiredness, or exhaustion	1079 (22.8)	151 (46.9)	928 (21.0)	<.001
Problems getting to sleep	1078 (22.8)	135 (41.9)	943 (21.4)	<.001
Unrefreshing sleep	770 (16.3)	115 (35.7)	655 (14.8)	<.001
Muscle aches or muscle pains	515 (10.9)	95 (29.5)	420 (9.5)	<.001
Pain in joints	351 (7.4)	84 (26.1)	267 (6.0)	<.001
Difficulty thinking or concentrating	293 (6.2)	97 (30.1)	196 (4.4)	<.001
Forgetfulness or memory problems	227 (4.8)	77 (23.9)	150 (3.4)	<.001
Dizziness or fainting	209 (4.4)	53 (16.5)	156 (3.5)	<.001

Abbreviations: COPD, chronic obstructive pulmonary disease; GED, General Educational Development; ME/CFS, myalgic encephalomyelitis/chronic fatigue syndrome–like illness.

^a^ Myalgic encephalomyelitis/chronic fatigue syndrome would not be identified during acute illness, but the index time was included along with all other time points for the dichotomous classification of ever vs never having ME/CFS.

^b^ Other gender included gender nonconforming, not listed, or preferred not to answer.

^c^ Other races listed in free-text responses entered by participants are included in eAppendix 3 in Supplement 1.

^d^ Hospitalization for the index illness was a new question added to the 3-month survey after April 14, 2021. There were 1134 participants missing a response to this question that were included as a separate category in the analysis.

^e^ Viral prevalence greater than 50% was used to determine the dominant variant.

^f^ Any type of tobacco, including smokeless tobacco.

^g^ Included coronary artery disease, heart failure, and cardiomyopathy.

^h^ Other conditions listed in free-text responses entered by participants that could contribute to ME/CFS symptoms. A list of the free-text entries is included in eAppendix 2 in Supplement 1.

The weighted percentage of participants meeting the ME/CFS criteria at 3 months was 3.4% in the COVID-19–positive group and 3.7% in the COVID-19–negative group, and there was no statistically significant difference between the COVID-19–positive and COVID-19–negative groups in the prevalence of ME/CFS at any time point through 12 months of follow-up (range, 2.8%-3.7% in the COVID-19–positive group and 3.1%-4.5% in the COVID-19–negative group) (Table 3). At each follow-up survey, approximately one-third of the COVID-19–positive group and one-third of the COVID-19–negative group (range, 31.0%-37.6%) reported 1 or more of the 5 ME/CFS symptoms assessed. In both the COVID-19–positive group and the COVID-19–negative group, unrefreshing sleep was the most frequently reported ME/CFS symptom (range, 20.2%-26.3%), followed by postexertional malaise (range, 16.9%-22.4%) and orthostatic intolerance (range, 9.0%-13.1%).

**Table 3.** Observed and Weighted ME/CFS Outcomes Across Time by COVID-19 Groups^a^

ME/CFS criterion	Participants, %							
	COVID-19 positive				COVID-19 negative			
	3 mo	6 mo	9 mo	12 mo	3 mo	6 mo	9 mo	12 mo
**Observed results**								
ME/CFS-like illness^b^	2.7	1.9	2.6	2.6	3.8	3.2	3.7	3.6
Reported individual criteria								
Postexertional malaise	17.2	15.4	16.0	16.3	20.1	19.2	21.5	24.4
Unrefreshing sleep	20.9	21.4	20.6	22.2	22.8	23.2	25.0	25.2
Fatigue	9.2	8.0	9.3	9.4	10.1	9.8	12.0	12.7
Orthostatic intolerance	5.4	4.8	5.7	5.9	8.8	8.6	9.2	9.6
Cognitive impairment	1.3	1.1	1.5	1.4	3.5	2.2	2.1	2.5
Criteria met, No.								
0	69.6	70.0	70.6	69.5	65.4	66.6	64.2	59.1
1	15.9	17.0	15.3	15.5	17.6	16.4	17.2	22.0
2	8.4	7.5	7.7	8.3	8.6	8.8	8.9	9.1
3	3.2	3.4	3.7	4.1	4.4	4.6	5.8	5.7
4	2.5	1.8	2.3	2.1	2.6	3.0	3.4	3.2
5	0.4	0.2	0.4	0.4	1.3	0.7	0.8	0.9
**Weighted results^a^**								
ME/CFS-like illness^b^	3.4	2.8	3.6	2.8	3.7	3.1	4.4	4.5
Reported individual criteria								
Postexertional malaise	18.5	16.9	17.9	17.3	18.9	18.6	20.8	22.4
Unrefreshing sleep	22.4	22.9	22.8	23.4	20.2	24.3	26.3	22.6
Fatigue	6.5	6.1	7.3	6.2	8.2	8.3	9.2	8.7
Orthostatic intolerance	10.3	9.0	10.6	10.4	9.6	9.4	13.1	12.9
Cognitive impairment	1.4	1.2	1.8	1.6	3.3	2.4	2.2	2.0
Criteria met, No.								
0	67.9	68.3	68.5	68.1	69.0	65.9	63.7	62.4
1	16.1	17.2	15.0	15.9	15.5	17.8	17.2	20.8
2	8.9	7.8	8.4	8.7	7.6	8.9	8.9	8.2
3	3.5	3.8	4.3	4.5	4.2	3.9	5.1	4.0
4	3.2	2.7	3.1	s	2.1	2.3	4.1	3.8
5	0.4	0.3	0.6	0.6	1.7	1.3	0.9	0.9

Abbreviation: ME/CFS, myalgic encephalomyelitis/chronic fatigue syndrome–like illness.

^a^ Weighted by inverse propensity scores.

^b^ Defined as meeting criteria for postexertional malaise, unrefreshing sleep, and fatigue and for either orthostatic intolerance or cognitive impairment.

Table 4 summarizes the time course of symptoms in the groups that ever or never had ME/CFS symptoms stratified by COVID-19 status. The group that ever had ME/CFS symptoms consistently rated their symptoms as more severe (ie, higher scores) compared with the group that never had ME/CFS symptoms regardless of COVID-19 status. The effect size for the difference in scores between participants who ever and never had ME/CFS symptoms for both the COVID-19–positive group and the COVID-19–negative group was moderate to very large for nearly all symptoms at all time points, suggesting greater symptom prevalence and severity among the group that ever had ME/CFS symptoms. The effect sizes tended to be larger for the COVID-19–positive group (Cohen *d* range, 2.72 [95% CI, 2.72-8.05] to 0.10 [95% CI, 0.06-0.22]) compared with the COVID-19–negative group (Cohen *d* range, 1.46 [95% CI, 1.45-4.30] to 0.25 [95% CI, 0.05-0.34]).

**Table 4.** Post–Acute Illness Health Status by COVID-19 Group and Ever Having ME/CFS-Like Illness, Weighted by Inverse Propensity Scores

Health status	COVID-19 status, ME/CFS-like illness status															
	3 mo				6 mo				9 mo				12 mo			
	Positive		Negative		Positive		Negative		Positive		Negative		Positive		Negative	
	Ever	Never	Ever	Never	Ever	Never	Ever	Never	Ever	Never	Ever	Never	Ever	Never	Ever	Never
Weighted total participants, No.^a^	100	1740	96	1633	88	1582	104	1537	80	1178	90	1251	58	855	77	927
Symptom severity score, mean (SD)^b^																
Difficulty thinking or concentrating that caused you to substantially cut back on your activities	9.6 (3.5)	6.3 (3.3)	11.3 (6.1)	5.8 (5.5)	10.4 (4.0)	5.9 (3.2)	9.9 (7.6)	5.7 (5.9)	5.1 (3.8)	3.5 (2.8)	4.5 (5.0)	3.2 (5.2)	10.3 (3.7)	6.0 (3.3)	11.4 (7.0)	5.7 (4.8)
Forgetfulness or memory problems that caused you to substantially cut back on your activities	9.1 (3.7)	5.9 (3.3)	11.2 (6.2)	6.6 (6.7)	10.6 (4.0)	5.3 (3.1)	10.3 (8.2)	6.5 (5.6)	11.3 (3.5)	6.4 (3.4)	11.2 (6.2)	7.0 (6.8)	9.8 (3.5)	5.8 (3.3)	11.0 (7.5)	6.4 (5.2)
Dizziness or fainting	3.8 (3.5)	3.5 (2.9)	8.4 (7.1)	4.2 (4.3)	4.8 (3.6)	2.8 (2.2)	8.6 (8.5)	3.3 (3.4)	9.4 (3.6)	5.9 (3.3)	8.0 (5.6)	6.4 (6.2)	5.1 (3.8)	3.5 (2.8)	4.5 (5.0)	3.2 (5.2)
Fatigue, tiredness, or exhaustion	10.1 (3.4)	5.5 (3.0)	10.8 (5.5)	5.8 (5.3)	10.5 (3.6)	5.6 (3.0)	11.8 (5.2)	5.4 (5.6)	11.1 (3.9)	5.7 (3.0)	11.4 (6.7)	6.4 (5.8)	10.6 (3.4)	5.8 (3.1)	11.3 (4.9)	5.5 (5.1)
Muscle aches or muscle pains	8.8 (4.0)	5.0 (3.0)	9.1 (6.4)	5.3 (6.0)	9.6 (4.1)	5.3 (2.9)	10.4 (6.7)	4.0 (5.2)	10.1 (3.5)	5.9 (3.0)	11.3 (5.3)	5.7 (5.8)	9.6 (3.5)	4.9 (3.1)	7.7 (8.0)	5.7 (5.3)
Pain in joints	10.3 (4.4)	5.5 (3.3)	9.3 (7.6)	6.6 (6.7)	10.1 (3.6)	5.7 (3.1)	8.5 (7.7)	5.2 (5.5)	10.1 (3.4)	5.7 (3.1)	10.9 (5.9)	6.8 (6.1)	9.1 (4.0)	6.1 (3.4)	8.4 (4.5)	6.0 (4.9)
Problems getting to sleep, sleeping through the night, or waking up on time	9.9 (3.6)	6.6 (3.4)	10.8 (5.5)	6.3 (6.0)	9.6 (3.3)	6.5 (3.5)	10.6 (7.1)	6.7 (5.7)	9.6 (3.5)	4.9 (3.1)	7.7 (8.0)	5.7 (5.3)	11.2 (3.2)	6.5 (3.2)	10.6 (6.1)	6.5 (5.8)
Unrefreshing sleep	10.0 (3.5)	5.8 (3.2)	9.9 (5.9)	5.8 (5.9)	9.7 (3.5)	5.9 (3.3)	11.9 (6.6)	5.7 (5.5)	11.5 (4.1)	6.1 (3.0)	10.0 (9.3)	7.1 (5.7)	10.1 (3.1)	5.8 (3.1)	9.0 (6.7)	6.0 (5.5)
ME/CFS-like illness diagnostic criteria, No. (%)^c^																
Postexertional malaise	87 (85.0)	267 (14.8)	79 (81.1)	260 (15.1)	67 (72.6)	218 (13.5)	84 (79.6)	231 (14.6)	66 (82.6)	162 (13.5)	75 (84.3)	207 (16.1)	43 (73.7)	118 (13.5)	66 (84.7)	161 (16.8)
Unrefreshing sleep	83 (81.0)	343 (18.9)	76 (77.3)	300 (17.3)	76 (81.9)	312 (19.2)	74 (70.1)	340 (21.5)	66 (81.7)	225 (18.7)	79 (87.8)	283 (22.0)	49 (83.9)	172 (19.7)	55 (70.4)	173 (18.1)
Fatigue	74 (73.0)	47 (2.6)	71 (73.0)	71 (4.1)	56 (60.8)	48 (2.9)	85 (80.1)	52 (3.3)	56 (69.5)	37 (3.1)	70 (77.8)	53 (4.1)	31 (54.1)	26 (2.9)	51 (66.3)	39 (4.1)
Orthostatic intolerance	10 (9.6)	20 (1.1)	36 (37.1)	21 (1.2)	12 (13.0)	8 (0.5)	28 (26.6)	13 (0.8)	10 (12.7)	13 (1.1)	18 (20.3)	12 (0.9)	7 (11.3)	8 (0.9)	12 (15.5)	8 (0.8)
Cognitive impairment	75 (73.7)	123 (6.8)	75 (77.4)	96 (5.5)	66 (70.6)	88 (5.4)	67 (62.9)	91 (5.7)	55 (68.2)	78 (6.5)	67 (75.3)	109 (8.4)	36 (62.0)	60 (6.8)	51 (66.0)	78 (8.2)
PROMIS-29 domain T scores, mean (SD)																
Higher score is better																
Cognitive function	33.6 (6.3)	48.9 (8.7)	32.5 (13.6)	47.5 (15.2)	33.3 (7.3)	49.5 (8.7)	34.1 (12.7)	48.7 (15.3)	32.9 (6.6)	49.2 (8.9)	29.9 (11.2)	48.6 (15.3)	33.0 (6.4)	49.5 (9.1)	31.3 (12.2)	48.4 (16.4)
Physical function	36.7 (5.8)	52.3 (5.7)	36.7 (8.2)	51.4 (11.5)	37.1 (6.3)	52.4 (5.7)	36.4 (10.5)	51.4 (11.6)	37.9 (5.7)	52.2 (5.8)	34.9 (8.4)	51.2 (11.3)	38.6 (5.3)	52.1 (6.0)	36.2 (9.8)	50.6 (11.8)
Social participation	38.7 (8.1)	55.9 (7.5)	38.5 (10.5)	54.4 (14.0)	39.1 (8.4)	56.6 (7.2)	38.3 (14.1)	55.0 (13.9)	41.2 (6.6)	56.6 (7.2)	36.1 (13.0)	55.0 (12.9)	42.8 (6.5)	57.0 (6.9)	36.2 (14.0)	54.7 (13.6)
Lower score is better																
Anxiety	62.4 (8.1)	51.3 (7.7)	61.5 (14.0)	53.1 (13.5)	62.5 (8.4)	50.9 (7.6)	59.8 (15.0)	52.3 (13.3)	61.1 (8.0)	50.6 (7.8)	59.5 (14.4)	51.8 (14.0)	62.2 (8.5)	50.2 (7.9)	60.3 (10.5)	51.5 (14.0)
Depression	59.0 (8.2)	49.0 (7.0)	57.4 (15.1)	49.9 (12.4)	59.0 (8.7)	48.7 (7.0)	61.0 (13.1)	49.5 (12.7)	58.9 (7.9)	48.7 (7.0)	60.1 (13.7)	49.4 (12.7)	59.8 (9.0)	48.4 (7.0)	57.4 (16.2)	49.4 (13.1)
Fatigue	64.9 (6.7)	50.3 (8.1)	66.3 (11.7)	51.8 (14.2)	65.5 (6.9)	49.9 (8.2)	66.7 (14.5)	51.3 (14.5)	64.5 (6.5)	49.8 (8.5)	68.9 (9.4)	51.4 (14.1)	64.8 (6.4)	49.4 (8.6)	67.4 (12.2)	50.8 (14.6)
Pain interference	62.4 (8.0)	46.4 (6.2)	62.6 (12.3)	48.4 (12.7)	62.7 (7.9)	46.5 (6.3)	63.3 (12.8)	48.2 (12.2)	61.4 (7.9)	46.9 (6.6)	64.5 (13.1)	48.4 (12.5)	61.8 (7.2)	46.7 (6.5)	62.6 (11.9)	48.9 (13.1)
Sleep disturbance	56.1 (3.8)	50.8 (3.6)	55.7 (5.7)	51.2 (6.7)	55.2 (4.2)	50.7 (3.6)	56.4 (5.8)	51.0 (7.0)	55.9 (4.0)	50.5 (3.7)	57.1 (4.6)	51.2 (6.7)	55.7 (3.7)	50.6 (3.9)	55.7 (6.4)	50.9 (6.3)

Abbreviations: ME/CFS, myalgic encephalomyelitis/chronic fatigue syndrome–like illness; PROMIS-29, Patient-Reported Outcomes Measurement Information System–29.

^a^ Nonmissing weighted total. The number of missing cases was 3 or fewer across all groups and time points.

^b^ Symptoms were assessed using the Centers for Disease Control and Prevention (CDC) ME/CFS Symptom Screener.

^c^ Percentages were calculated among nonmissing cases. The number of missing cases was less than 10 across all groups and time points.

In weighted and adjusted results, there were no statistically significant differences in the odds of ME/CFS between COVID-19–positive and COVID-19–negative participants at any time point (marginal odds ratio range, 0.84 [95% CI, 0.42-1.67] to 1.18 [95% CI, 0.55-2.51]) (Figure). The odds of ME/CFS also did not significantly differ across time points in either the COVID-19–positive group or the COVID-19–negative group (Figure). The marginal incidence rate ratio (MIRR) of ME/CFS symptom count was not significantly different between the COVID-19–positive and COVID-19–negative groups at any time point (eg, 12-month MIRR: 0.96; 95% CI. 0.74-1.24) (Figure) and was not significantly different over time in the COVID-19–positive or COVID-19–negative group. Results were consistent when using a propensity score–matched sample (eFigure 3 in Supplement 1) compared with the full sample with IPW.

**Figure.**
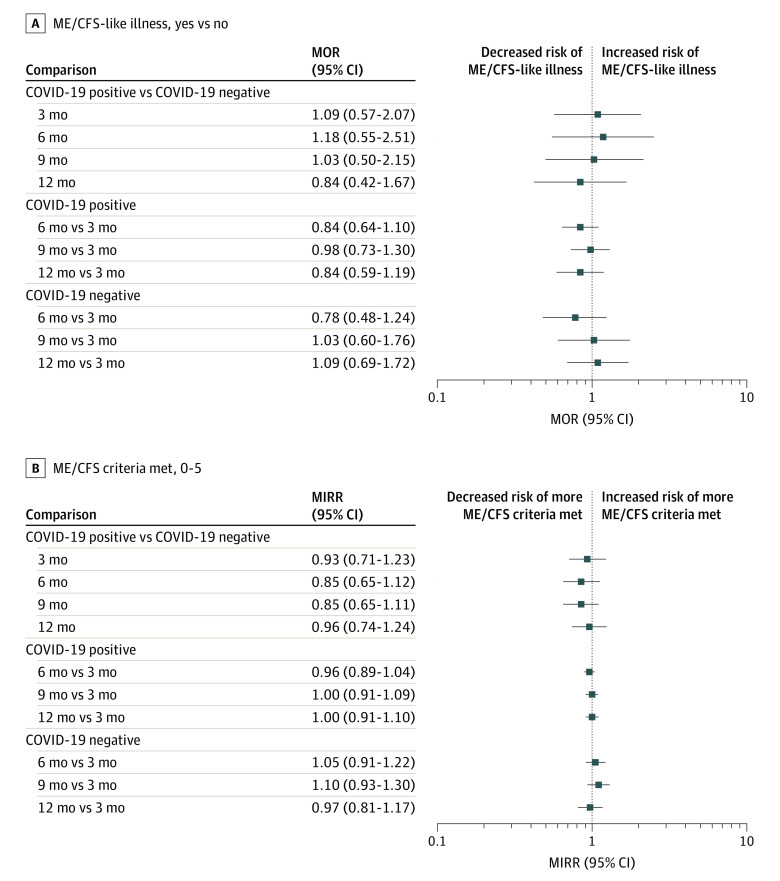
Weighted Marginal Effect Estimates for the Association Between Index COVID-19 Status and Myalgic Encephalomyelitis/Chronic Fatigue Syndrome (ME/CFS) Outcomes MIRR indicates marginal incidence rate ratio; MOR, marginal odds ratio.

## Discussion

Our findings suggest that COVID-19 is no more likely than other acute infections to be associated with ME/CFS and that acute illnesses more broadly may be associated with chronic symptom burden from ME/CFS. The odds of meeting ME/CFS criteria at quarterly follow-up time points through 12 months did not differ between the COVID-19–positive and COVID-19–negative groups, and prevalence was relatively stable over this time in both groups (range, 2.8%-4.5%). Additionally, symptom severity scores for participants meeting ME/CFS criteria were similar in both COVID-19 groups and were significantly greater than for those not meeting ME/CFS criteria. The high symptom burden for participants meeting ME/CFS criteria persisted through 12 months in both cohorts, emphasizing the potential for a long duration of illness and disability. It is also important to note that the prevalence of individual symptoms, such as postexertional malaise and sleep problems, was higher than the full complex of ME/CFS symptoms and may also impose a substantial burden to patients. These findings emphasize the importance of developing clinical management strategies for patients with post–acute infection syndromes.

Our study design required an acute infection prompting COVID-19 testing. While participants testing positive had an identifiable infection (SARS-CoV-2), we were unable to collect data on the specific infection that led to symptoms from those testing negative. Our finding that ME/CFS-like illness was equally likely to occur after SARS-CoV-2 and unknown infection is similar to findings in a prospective, population-based study of acute respiratory illness in adults in the UK that found symptom burdens to be similar among participants with and without prior SARS-CoV-2 infection.^20^ In contrast, a large retrospective analysis of electronic health records conducted in the UK in 2020 suggested that SARS-CoV-2 infection resulted in significantly more postacute symptoms than did influenza, although symptoms were also common following influenza.^21^ However, that study’s definition of ME/CFS was reliant on symptom documentation in electronic health records, which can vary based on clinician expectations and inquiry about postinfectious symptoms in patients with SARS-CoV-2 infection compared with influenza.

Increasingly, ME/CFS is recognized as a chronic sequela of a variety of infections. A recently published 17-year population-based cohort study (2000-2017) using data from the Taiwan National Health Insurance Research Database demonstrated an elevated risk of CFS following infection with varicella-zoster virus, *Mycobacterium tuberculosis*, *Escherichia coli*, *Staphylococcus aureus*, influenza virus, and *Borrelia burgdorferi*.^22^ The prevalence of ME/CFS-like illness in the present study sample (≤4.5%) was lower than that in several previous studies of ME/CFS following a specific infection or syndrome (ie, 27% in a 4-year follow-up study of 223 patients who experienced severe acute respiratory syndrome,^23^ 13% in a 6-month follow-up study of 301 adolescents diagnosed with acute Epstein-Barr virus infection^24^). Study differences, such as population sample (eg, community, clinic, or tertiary care), methods of case ascertainment, case definition, and differences in the infection preceding ME/CFS could account for this. Given that there are over 100 million confirmed cases of COVID-19 illness in the US,^25^ the prevalence of ME/CFS-like illness in our study represents a high absolute proportion of individuals possibly affected with ME/CFS associated with COVID-19 (2.8-3.6 million individuals).

While there are limited published data on the prevalence of post–COVID-19 ME/CFS, our findings are similar to the prevalence of 2.5% recently reported in a 6-month follow-up study of 120 patients with COVID-19 hospitalized at a university-affiliated hospital in Tehran, Iran,^26^ and a cross-sectional survey of 437 participants conducted in Jordan that found 2.8% of the sample to have COVID-19–related ME/CFS.^27^ Given that our study included a large, diverse array of participants recruited primarily from ambulatory testing and community settings, we expected somewhat lower prevalence than reported in cohorts recruited from acute care settings where initial illnesses may have been more severe. Our estimates may underrate the potential burden of ME/CFS among those who were hospitalized or experienced substantial morbidity from their COVID-19 illness. The severity of ongoing illness may also have affected participants’ willingness to volunteer for our study.

Despite the low overall prevalence of ME/CFS-like illness, we observed a high individual prevalence of ME/CFS symptoms. At each follow-up, approximately one-third of participants in both the COVID-19–positive group and the COVID-19–negative group reported 1 or more of the 5 symptoms included in the IOM definition of ME/CFS. In both groups, unrefreshing sleep was most frequently reported (range, 20.2%-26.3%) followed by postexertional malaise (range, 16.9%-22.4%) and orthostatic intolerance (range 9.0%-13.1%). The IOM criteria for ME/CFS are based on the combination of core symptoms that best differentiate the illness from other diagnoses; however, in the absence of complete clinical evaluation, classification based only on symptoms cannot rule out other conditions that contribute to the illness experienced by participants. The similarities in symptoms between the COVID-19–positive and COVID-19–negative participants support the suggestion that the clinical approach to postacute infection syndromes would be similar and care for these patients could be managed in a similar clinical setting, contributing to efficiency.

### Strengths and Limitations

Our study has several strengths. We conducted an in-depth study of ME/CFS after an acute index illness during the COVID-19 pandemic in a population that included both COVID-19–positive and COVID-19–negative individuals. By including COVID-19–negative individuals who presented with another acute infection–like illness, we could assess the differential risk of ME/CFS following SARS-CoV-2 infection compared with other uncharacterized acute illnesses. We collected standardized and detailed self-reported symptom data at multiple time points. Symptom-based illnesses are often not recognized, and clinicians may inaccurately or unsystematically code them in electronic medical records^28^; thus, studies using data from electronic health records lacking patient-reported data are inadequate to understand the epidemiology of SARS-CoV-2–associated postacute syndromes. Other strengths include the large sample size from multiple, diverse geographic locations and settings (eg, ambulatory, emergency, and inpatient) across the US and inclusion of English and Spanish speakers. Our use of a robust IPW strategy helped mitigate the impact of significant baseline differences between COVID-19–positive and COVID-19–negative groups when evaluating the association of COVID-19 status with ME/CFS.

Our study also has limitations. Differences in baseline characteristics between COVID-19–positive and COVID-19–negative groups may not have been fully mitigated despite the robust IPW strategy. In particular, new SARS-CoV-2 infections were more common in the COVID-19–negative group, and some individuals may have been unaware of a subsequent infection. New infection was used as an adjustment factor, mitigating but not eliminating its impact. In both groups, symptoms suggestive of ME/CFS were reported prior to the index illness, but information was not complete enough to identify preexisting ME/CFS-like illness. This could have led to misclassification of ME/CFS-like illness in that we may have attributed symptoms to the index illness when those symptoms may have predated the illness. Furthermore, as ME/CFS is a symptom-based diagnosis, assessment of ME/CFS relies on self-reported symptoms that may be subject to recall and reporting bias. We used standardized questionnaires to assess symptoms to mitigate this problem. The specific infections or etiology for the acute symptoms in the COVID-19–negative group were not documented and likely heterogeneous. Some participants classified as having ME/CFS may have had an alternative diagnosis to explain their ME/CFS symptoms. To track the ME/CFS symptoms at early time points following the index illness, we did not consider 6-month duration in our ME/CFS algorithm, as would be used clinically. Less than one-third of all respondents (28.6%) provided data at all follow-up time points, so we were unable to fully evaluate stability of ME/CFS symptoms over time. However, the use of GEE allowed population average estimates to be generated at each time point with all available data included.

False-positive and false-negative COVID-19 test results at enrollment may have led to misclassification in the cohorts.^29^ Although we adjusted for reports of subsequent COVID-19 illness in our main model, it is possible that not everyone with a subsequent illness was symptomatic or was tested for COVID-19; thus, we may have underreported the frequency of these events. Additionally, we did not collect histories of other infection or diagnostic tests and thus could not characterize the specific infection that might account for ME/CFS in the COVID-19–negative group. The requirement for access to a verifiable COVID-19 test and internet-enabled device to complete surveys may have biased the sample to a more engaged and technologically savvy population. Furthermore, we did not include vaccination status in our analysis.

## Conclusions

In this prospective, multicenter cohort study of participants with acute infection–like symptoms prompting SARS-CoV-2 testing, the prevalence of ME/CFS symptoms was similar between COVID-19–positive and COVID-19–negative individuals. At 12 months, 2.8% of those in the COVID-19–positive group and 4.5% in the COVID-19–negative group met the definition of ME/CFS. Our findings suggest that ME/CFS may follow several precipitating events (acute COVID-19 illness, other acute infections, or life disruptions due to the COVID-19 pandemic) but that regardless of reason or exact percentages, there will be millions affected who will seek care.

## Notes

Supplement 1. **eFigure 1.** Flowchart of INSPIRE Study Sample **eAppendix 1.** CDC ME/CFS Symptom Screener–Short Form, Version 1.2 **eTable 1.** Operationalized Algorithm for the 2015 IOM ME/CFS Case Definition **eAppendix 2.** List of Conditions Potentially Contributing to ME/CFS Symptoms Entered by Participants **eAppendix 3.** Other Races Entered by Participants **eTable 2.** Observed Distribution of Confounders and Covariates Between COVID-19 Groups **eFigure 2.** Absolute Standardized Differences Between COVID-19 Groups **eFigure 3.** Marginal Effects of Index COVID-19 Status on the ME/CFS Outcomes Based on Matched Sample

Supplement 2.
Innovative Support for Patients with SARS-CoV-2 Infections Registry (INSPIRE) Group

Supplement 3.
Data Sharing Statement
