# Supplementary material for: Myalgic Encephalomyelitis/Chronic Fatigue Syndrome After SARS-CoV-2 Infection
Source: JAMA Netw Open. 2024 Jul 24;7(7):e2423555. doi: 10.1001/jamanetworkopen.2024.23555 (PMC11270135; doi:10.1001/jamanetworkopen.2024.23555)
Supplement: Supplement 1. — eFigure 1. Flowchart of INSPIRE Study Sample eAppendix 1. CDC ME/CFS Symptom Screener–Short Form, Version 1.2 eTable 1. Operationalized Algorithm for the 2015 IOM ME/CFS Case Definition eAppendix 2. List of Conditions Potentially Contributing to ME/CFS Symptoms Entered by Participants eAppendix 3. Other Races Entered by Participants eTable 2. Observed Distribution of Confounders and Covariates Between COVID-19 Groups eFigure 2. Absolute Standardized Differences Between COVID-19 Groups eFigure 3. Marginal Effects of Index COVID-19 Status on the ME/CFS Outcomes Based on Matched Sample [file jamanetwopen-e2423555-s001.pdf]

Supplementary Online Content

Unger ER, Lin JMS, Wisk LE, et al; INSPIRE Group. Myalgic encephalomyelitis/chronic fatigue syndrome after SARS-CoV-2 infection. *JAMA Netw Open*. 2024;7(7):e2423555. doi:10.1001/jamanetworkopen.2024.23555

eFigure 1. Flowchart of INSPIRE Study Sample

eAppendix 1. CDC ME/CFS Symptom Screener–Short Form, Version 1.2

eTable 1. Operationalized Algorithm for the 2015 IOM ME/CFS Case Definition

eAppendix 2. List of Conditions Potentially Contributing to ME/CFS Symptoms Entered by Participants

eAppendix 3. Other Races Entered by Participants

eTable 2. Observed Distribution of Confounders and Covariates Between COVID-19 Groups

eFigure 2. Absolute Standardized Differences Between COVID-19 Groups

eFigure 3. Marginal Effects of Index COVID-19 Status on the ME/CFS Outcomes Based on Matched Sample

This supplementary material has been provided by the authors to give readers additional information about their work.

© 2024 Unger ER et al. *JAMA Network Open*.

eFigure 1. Flowchart of INSPIRE Study Sample (December 11, 2020 through August 29, 2022)

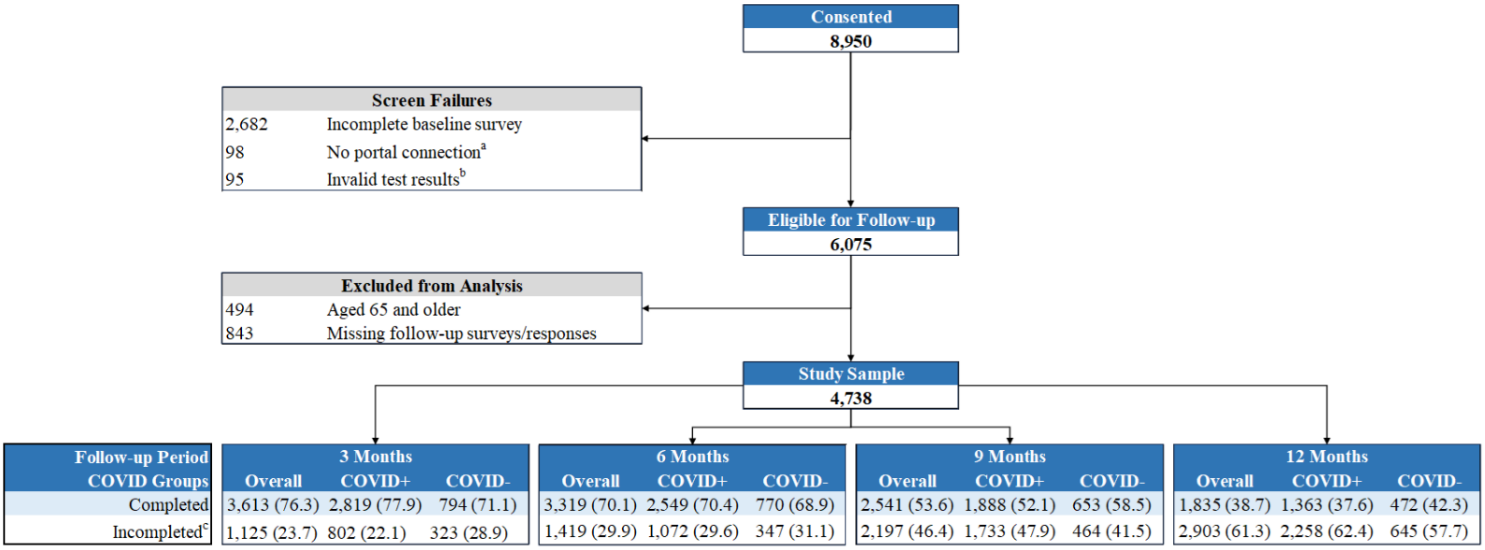

[a] Portal connection was requirement for follow-up eligibility from study start through 3/21/22. Portal connection means ever connected EHR, clinical and/or pharmacy portal with Hugo Health.
[b] Invalid covid test results = no proof of test or had a positive test >42 days before enrollment.
[c] Incomplete surveys due to withdrawal, deceased, skipping, and end-of-study censoring by 2/28/2023.
NOTE: 532 respondents completed only 1 survey; 1,126 completed only 2 surveys; 1,032 completed only 3 surveys; 1,357 completed all 4 surveys.

© 2024 Unger ER et al. *JAMA Network Open*.

eAppendix 1. eCDC ME/CFS Symptom Screener – Short Form v1.2

SYMPTOMS

1. Below is a list of symptoms many individuals experience. Please tell us whether or not you have any of the symptoms listed below. FOR EACH SYMPTOM, PLEASE CIRCLE THE APPROPRIATE ANSWERS IN THE GRID BELOW.

	**DURING THE PAST MONTH, HAVE YOU HAD THIS SYMPTOM?** *PLEASE CIRCLE 1 OR 2. IF 1 IS SELECTED, THEN GO TO COLUMN A*		**COLUMN A** **PRIOR TO THIS PAST MONTH, FOR HOW LONG HAD YOU EXPERIENCED THIS SYMPTOM?**		**COLUMN B** **DID YOU HAVE THIS SYMPTOM BEFORE THE RECENT HEALTH CONCERN THAT CAUSED YOU TO GET A COVID TEST?**	
	YES	NO	UNDER 6 MONTHS	6 MONTHS OR LONGER	YES	NO
Fatigue, tiredness, or exhaustion	1	2	1	2	1	2
Muscle aches/muscle pains	1	2	1	2	1	2
Pain in joints	1	2	1	2	1	2
Unrefreshing sleep	1	2	1	2	1	2
Problems getting to sleep, sleeping through the night, or waking up on time	1	2	1	2	1	2
Forgetfulness/memory problems that caused you to substantially cut back on your activities	1	2	1	2	1	2
Difficulty thinking or concentrating that caused you to substantially cut back on your activities	1	2	1	2	1	2
Dizziness or fainting	1	2	1	2	1	2

© 2024 Unger ER et al. *JAMA Network Open*.

SYMPTOMS (CONTINUED)

1A. For each symptom you noted in question 1, please fill in the grid to describe the frequency and the severity of the symptoms. FOR EACH SYMPTOM, PLEASE CIRCLE THE APPROPRIATE ANSWERS IN THE GRID BELOW.

SYMPTOMS	<u>DURING THE PAST MONTH</u>, HOW OFTEN HAVE YOU HAD THIS SYMPTOM?					<u>DURING THE PAST MONTH</u>, HOW BAD WAS THIS SYMPTOM?				
	A LITTLE OF THE TIME	SOME OF THE TIME	A GOOD BIT OF THE TIME	MOST OF THE TIME	ALL OF THE TIME	VERY MILD	MILD	MODERATE	SEVERE	VERY SEVERE
Fatigue, tiredness or exhaustion	1	2	3	4	5	1	2	3	4	5
Muscle aches/muscle pains	1	2	3	4	5	1	2	3	4	5
Pain in joints	1	2	3	4	5	1	2	3	4	5
Unrefreshing sleep	1	2	3	4	5	1	2	3	4	5
Problems getting to sleep, sleeping through the night, or waking up on time	1	2	3	4	5	1	2	3	4	5
Forgetfulness/memory problems that caused you to substantially cut back on your activities	1	2	3	4	5	1	2	3	4	5
Difficulty thinking or concentrating that caused you to substantially cut back on your activities	1	2	3	4	5	1	2	3	4	5
Dizziness or fainting	1	2	3	4	5	1	2	3	4	5

© 2024 Unger ER et al. *JAMA Network Open*.

1B. For Fatigue symptom you noted in question 1, please answer the following questions.

1B.a When this fatigue, tiredness, or exhaustion began, would you say that it came on all of a sudden, or slowly over time?

- 1 All of sudden
- 2 Slowly over time
- 6 Not applicable
- 8 Don't know

1B.b In what month and year did your fatiguing illness begin?
Month_____ Year_____

1 B.c. When you are fatigued, does rest make your fatigue better?

- 1 Yes, a lot
- 2 Yes, a little
- 3 No, not very much
- 6 Not applicable
- 8 Don't know

1.B.d. When you are fatigued, has this fatigue substantially limited your ability to occupational, educational, social, or personal activities?

- 1 Yes
- 2 No
- 6 Not applicable
- 8 Don't know

1C. For the symptoms noted in question 1,
Do any of them get worse for at least 24 hours after you engage in activities (physical or mental) that you were used to doing with no problems?

- 1 Yes
- 2 No
- 6 Not applicable
- 8 Don't know

Frequency (how often) and intensity (severity) of each symptom were rated on a 5-point scale, ranging from 1 ("a little of the time") to 5 ("all of the time") for frequency and from 1 ("very mild") to 5 ("very severe") for intensity. Duration for each symptom was rated on a 2-point scale, ranging from 1 (less than 6 months) to 2 (6 months or longer). For each symptom, survey skip logic was used, so that a respondent who did not experience any given symptom had the frequency and intensity for that symptom set to 0 to indicate absence of frequency and intensity. To make the scoring comparable with the first version of the

© 2024 Unger ER et al. *JAMA Network Open*.

CDC ME/CFS Symptom Inventory, we recoded the frequency and intensity raw responses into equidistant responses before multiplication scoring (ie, frequency: 0 = "no symptom," 1 = "a little of the time," 2 = "some of the time," 3 = "a good bit of the time" or "most of the time," and 4 = "all of the time"; intensity: 0 = "no symptom," 1 = "very mild" or "mild," 2.5 = "moderate," and 4 = "severe" or "very severe"). We multiplied frequency by intensity responses to form the severity score for each symptom, which ranged from 0 (no symptom) to 16 (most severe). If respondents experienced any of these symptoms, response to 1C evaluated post-exertional malaise.

© 2024 Unger ER et al. *JAMA Network Open*.

eTable 1. Operationalized Algorithm for the 2015 IOM ME/CFS Case Definition

**1. A substantial reduction or impairment in the ability to engage in pre-illness level of activity (occupational, educational, social, or personal life)**	Substantial reduction or impairment measured by a T-score of <40 on the Physical Function (PF) subscale of the PROMIS-29 measure **accompanied by:**<br><br>Profound fatigue not relieved by rest, measured on the CDC ME/CFS Symptom Screener by “3=a good bit of the time”, “4=most of the time”, or “5=all the time” on frequency<br>**AND**<br>“3=moderate”, “4=severe”, or “5=very severe” on intensity for the symptom fatigue, tiredness, or exhaustion<br>**AND**<br>A response of either “no, not at all” or “no, not very much” to the follow-up item, “When you are fatigued, does rest make your fatigue better?”
**2. Post-exertional malaise (PEM)**	Measured by a “yes” response to the CDC ME/CFS Symptom Screener[a] item, “Do any of your symptoms get worse for at least 24 hours after you engage in activities (physical or mental) that you were used to doing with no problems?”
**3. Unrefreshing sleep (meeting one of the following two sleep-related response criteria from the CDC ME/CFS Symptom Screener[a])**	A 3 or higher on frequency **and** a 3 or higher on intensity of unrefreshing sleep<br>**OR**<br>A 3 or higher on frequency **and** a 3 or higher on intensity of problems getting to sleep, sleeping through the night, or waking up on time
**ME/CFS requires all three criteria above to be met. In addition, one of the following two criteria must be met.**	
**4a. Cognitive impairment (meeting one of the following two cognitive-related response criteria from the CDC ME/CFS Symptom Screener[a])**	A 3 or higher on frequency **and** a 3 or higher on intensity for one of the following symptoms: forgetfulness/memory problems **or** difficulty thinking/concentrating

© 2024 Unger ER et al. *JAMA Network Open*.

**4b. Orthostatic intolerance (OI) (meeting the following criteria from the CDC ME/CFS Symptom Screener[a])**	A 3 or higher on frequency **and** a 3 or higher on intensity for the symptom: dizziness/fainting

[a]The CDC ME/CFS Symptom Screener v1.2 is included in the supplement. The 2015 IOM case definition for ME/CFS[15] uses data from the following symptoms: (1) fatigue, tiredness, or exhaustion; (2) post-exertional malaise; (3) unrefreshing sleep, problems getting to sleep, sleeping through the night, or waking up on time; (4) cognitive impairment as indicated by forgetfulness/memory problems or difficulty thinking/concentrating; (5) orthostatic intolerance as indicated by dizziness/fainting.

© 2024 Unger ER et al. *JAMA Network Open*.

eAppendix 2. eLine List of Conditions Potentially Contributing to ME/CFS Symptoms Entered by Participants

Other: AML
Other: Addison's disease
Other: Addison's disease hypothyroid. Depression sleep apnea RLS
Other: Amyloidosis and Severe Neuropathy, Heart transplant in 2019
Other: Anemia
Other: Ankolosing Spondylitis
Other: Ankylosing Spondilitis
Other: Ankylosing Spondylitis
Other: Ankylosing spondylitis, ulcerative colitis
Other: Arthritis (ankylosing spondylitis)
Other: Auto immune conditions
Other: Autoimmune
Other: Autoimmune and Thyroid
Other: Autoimmune disease
Other: Autoimmune diseases cancer survivor
Other: Bi-polar
Other: Bilateral adrenal adenomas
Other: Bipolar 1 disorder
Other: Bipolar Disorder and ADHD
Other: Bipolar II disorder, moderate, depressed, with anxious distress
Other: Bipolar and had strep throat same week as covid the 2nd time
Other: Brain Cancer
Other: Brain cancer
Other: Brain tumor, likely acoustic neuroma
Other: Breast Cancer diagnosed 12/2019 takes tamoxifen daily
Other: Breast cancer
Other: Breast cancer 2007
Other: Breast cancer, migraine
Other: Brigades Syndrome
Other: Bronchiectisis, fibromyalgia, peripheral neuropathy, Barrett's esophagus
Other: CIDP, CROHNS
Other: CLL
Other: CML
Other: CROHN'S
Other: CTEPH
Other: Cancer
Other: Cancer de prostata tratado.
Other: Cancer history
Other: Cancer nodules in lungs
Other: Cancer survivor
Other: Celiac

© 2024 Unger ER et al. *JAMA Network Open*.

Other: Celiac Disease
Other: Celiac disease - Depression
Other: Celiac disease,Prefer not to answer
Other: Celiac, Lupus
Other: Chronic idiopathic angioedema, migraines, CRPS
Other: Chronic lymphocytic leukemia
Other: Chronic migraine
Other: Chronic migraines
Other: Chronic migraines, Major Depressive D/o (in remission)
Other: Colitis
Other: Connective tissue disease
Other: Continued chemotherapy treatment (in remission for AML)
Other: Crohn's
Other: Crohn's Disease
Other: Crohn's disease
Other: Crohn's disease, ADHD
Other: Crohn's, Ulcerative Colitis
Other: Crohns
Other: Crohns, wiskott Aldrich syndrome
Other: Crohn's
Other: Crohn's disease
Other: Currently pregnant
Other: Currently pregnant - due with twins July 4th
Other: Currently pregnant,Don't know
Other: DVT/PE
Other: Depression, fibromyalgia, low back pain
Other: Diagnosed with endometrial cancer on June 27. Surgery on July 14.
Other: Disseminated valley fever
Other: Dysautonomia, hypermobile Ehlers Danlos Syndrome, small fiber neuropathy
Other: EGPA
Other: Eczema, Chronic Migraines, PTSD, ADHD
Other: Endometriosis
Other: Endometriosis, Lyme disease, hashimoto thyroid, depression, anziety
Other: Epilepsy
Other: FND due to Covid
Other: Fibromalgia
Other: Fibromyalgia
Other: Fibromyalgia, chronic migraines
Other: Fibromyalgia, hashimoto
Other: Fibromyalgia, neuropathy
Other: Fibromylaga
Other: Follicular Lymphoma
Other: GRAVES DISEASE, OCCAISIONAL HEART PALPITATIONS

© 2024 Unger ER et al. *JAMA Network Open*.

Other: Gerd fibro,pcos,endometriosis
Other: Graves Disease
Other: Graves and rheumatoid
Other: Graves disease
Other: Graves, hyperlipidemia
Other: Gut issues, SIBO and IBS, gluten intolerance
Other: HIV
Other: HIV +
Other: HIV HEP B
Other: HIV+ Hep B+
Other: HIV/AIDS
Other: Hashimoto's
Other: Hashimoto's Thyroiditis
Other: Hashimotos
Other: Hashimotos Hypo Thyroidism
Other: Hashimotos disease
Other: Hashimotos thyroiditis, idiopathic anaphylaxis
Other: Hashimotos/Raynauds
Other: Hashimoto's Disease Celiac Disease
Other: Hashimoto's hypothyroidism
Other: Heart transplant & bilateral diaphragmatic paralysis
Other: High cholesterol Bipolar
Other: Hiv
Other: Hiv +
Other: Hyperthyroid/hashimotos
Other: Hypothyroidism, HIV, Arthritis
Other: Hypothyroidism, pregnancy
Other: Hypothyroidism; Vasculitis
Other: I have no Comorbidities but for Stage 4 breast cancer and taking iimmunosuppressing drugs
Other: I smoke weed & vape concentrated THC oil.
Other: I tÃ©rmites Iron deficiency anemia
Other: I'm 8mo pregnant
Other: IBD
Other: IBS
Other: IBS, generalized anxiety disorder, ADHD-Inattentive Type
Other: IIH
Other: ITP
Other: ITP Autoimmune
Other: Ibs, erosion of stomach
Other: Ibs, gerd
Other: Ibs, mvp, endmetrisos
Other: Immune comprimsed
Other: Immunodeficiency

© 2024 Unger ER et al. *JAMA Network Open*.

Other: Inactive Sarcoidosis
Other: Interstitial lung disease
Other: Iron deficiency
Other: Iron deficient anemia, PCOS
Other: Irritable Bowel Syndrome,Don't know
Other: LUPUS
Other: Leukemia (cml)
Other: Liver transplant
Other: Lot better now after Liver transplant in 3/2018
Other: Lung cancer
Other: Lung transplant
Other: Lupus
Other: Lupus and MS
Other: Lupus hypothyroidism hashimoto
Other: Lupus, depression
Other: Lymphoma
Other: Lynch Syndrome
Other: M.S.
Other: MCAS, Dystonia
Other: MDD, GAD
Other: MPA Anca Vasculitis
Other: MS
Other: MS Sarciodosis
Other: MS and depression
Other: MULTIPLE SCLEROSIS
Other: Major depression that existed before covid diagnosis
Other: Mantle cell Lymphoma
Other: Marfan Syndrome
Other: Marfan syndrome, Sleep Apnea, Chronic PTSD/Depression/Anxiety
Other: Marijuana smoker
Other: Microadenoma of pituitary gland
Other: Migraine
Other: Migraine, ADHD
Other: Migraine, PCOS
Other: Migraine, hypothyroid, anxiety
Other: Migraines
Other: Mitochondrial disease
Other: Ms
Other: Multiple Myeloma
Other: Multiple Sclerosis
Other: Multiple Sclerosis and PCOS
Other: Multiple Sclerosis, Lyme Disease
Other: Multiple myeloma,Don't know

© 2024 Unger ER et al. *JAMA Network Open*.

Other: Multiple sclerosis
Other: Muscular Dystrophy; ADHD; Anxiety; Depression
Other: Myotonic muscular dystrophy
Other: Myriadâ€”orthopedic. Neurological, gastrointestinal
Other: MÃ©niÃ¨re's disease
Other: Narcolepsy
Other: Narcolepsy w/ Cataplexy
Other: Neuroendocrine cancer
Other: Neurological and GI
Other: New onset anemia
Other: Non-hodgkin Lymphoma
Other: OCD
Other: OSA, osteopenia
Other: Osteogenesis imperfecta, HIV
Other: Osteopenia, iron deficiency-anemia, heavy menses occassionally
Other: PCOS
Other: PCOS, Depression/Anxiety, possible PTSD, Mediterranean Thalassemia
Other: PCOS,Prefer not to answer
Other: PMR
Other: POTS
Other: Pagets disease
Other: Pancreatic Cancer
Other: Parkinson's
Other: Paroxysmal Atrial Tachycardia; Sleep Apnea
Other: Pcos
Other: Polyarteritis Nodosa
Other: Polycystic Ovarian Syndrome
Other: Polycystic ovaries
Other: Polymyalga rheumatica
Other: Post partum gave birth 3/2/22
Other: Postural Orthostatic Tachycardia Syndrome (high functioning)
Other: Postural orthostatic tachycardia syndrome
Other: Pregnancy
Other: Pregnancy (12 weeks)
Other: Pregnancy, hypothyroidism
Other: Pregnant
Other: Pregnant, with gestational diabetes
Other: Premature ventricular contractions, sleep apnea
Other: Primary Lateral Sclerosis
Other: Progressive osseous heteroplasia
Other: Prostate Cancer
Other: Prostate cancer
Other: Psoriasis

© 2024 Unger ER et al. *JAMA Network Open*.

Other: Psoriatic arthritis
Other: Pulmonary Fibrosis
Other: Pulmonary embolism
Other: RA , Sjogren's, lung disease (etiology unknown), lymphoma (remission)
Other: RA, pulmonary fibrosis
Other: RA/ Lupus
Other: RHEUMATOID ARTHRITIS
Other: Ra
Other: Raynards Disease
Other: Raynauds
Other: Raynauds, Basal and Squamous cell skin cancer
Other: Recently diagnosed with likely thyroid cancer, also getting tx for carpal tunnel
Other: Recovering from cancer
Other: Reinodes
Other: Remission 6 years from brain cancer
Other: Rheumatoid arthritis
Other: Rheumatoid Arthritis
Other: Rheumatoid arthritis
Other: Rheumatoid arthritis (biologics)
Other: Rheumatoid arthritis,Prefer not to answer
Other: Rheumatoid arthritis/fibromyalgia
Other: SLE
Other: Sarcoidosis
Other: Sarcoidosis, hypothyroidism, PE
Other: Seizure Disorder and migraine headaches
Other: Severe Sleep Apnea
Other: Shingles
Other: Sjogrens, PPFE lung disease
Other: Sleep Apnea
Other: Sleep Apnea, Atrial Fibrillation
Other: Sleep Apnea, Dysthymia
Other: Sleep Apnea, Elevated Cholesterol, Severe Osteoarthritis both knees
Other: Sleep Apnia
Other: Sleep apnea
Other: Smoking marijuana
Other: Spinal cord injury
Other: Stomach cancer
Other: Systemic Lupus
Other: Systemic Lupus, Fibromyalgia, Psoriasis, POTS
Other: TOS, CHRONIC PAIN
Other: TTP
Other: Tmjd
Other: Transplant recipient, clotting issues

© 2024 Unger ER et al. *JAMA Network Open*.

Other: Transverse Mylitis
Other: Tuberculosis
Other: Type 2 diabetes
Other: UC
Other: Ulcerative Colitis
Other: Ulcerative colitis
Other: Ulcerative colitis, adrenal insufficiency, depression, hypothyroidism
Other: Ulcerative colitus
Other: Undifferentiated connective tissue disease
Other: Vid D defic; Hypocalcemic
Other: Vitamin D deficiency
Other: Von willebrands disease. Migraines
Other: Wegners & EOE
Other: anemia
Other: appendectomy in december 2021; three spine injuries and need knee surgery, diagnosed with anxiety, depression and PTSD
Other: arthritis, hashimoto's
Other: autoimmune
Other: autoimmune; treated with rituxan.
Other: breast cancer
Other: bronchiestisis, interstitial lung disease
Other: celiac disease
Other: cervical stenosis, neuropathy
Other: chrones disease
Other: diverticulosis, silent heart attack, IBS, Sleep Apnea
Other: generalized anxiety disorder
Other: hiv
Other: ibd, ibs
Other: iron-deficiency
Other: lupus
Other: migraine
Other: migraines
Other: multiple myeloma
Other: multiple sclerosis
Other: multiple sclerosis, osteoporosis, lymes disease, rheumatoid arthritis, meniere's disease
Other: neuropathy
Other: pnh
Other: post lung transplant
Other: pregnancy
Other: pregnant
Other: pregnant, otherwise n/a
Other: prostate cancer
Other: psoriasis

© 2024 Unger ER et al. *JAMA Network Open*.

Other: pulmonary hypertension and Crohn's Disease

Other: rheumatoid arthritis

Other: sjogren's/lupus and connective tissue disorder

Other: sleep apnea

Other: too much to write but main one is Lupus. After COVID-19 in September, had mini strokes, and ongoing other problems

Other: ulcerative colitis

Other: von willerbrand disease and vasal vagal syncope

© 2024 Unger ER et al. *JAMA Network Open*.

eAppendix 3. eList of Other Races Entered by Participants[a]

3 or more Eastern European countries
51 percent European/39 percent Native American genes.
Afghan
Afrolatino
American
Arab
Armenian
Armenian / Salvadorian
Armenian. (kinda "white")
Ashkenazi
Ashkenazi Jew
Ashkenazi Jewish
Asian American
BRAZILIAN
Belgian-Tunisian
Bi Racial
Biracial
Black Honduran
Black white native
Blend of Black,Spanish and Native
Brazilian
Brown
Brown!
Caribbean
Caribbean America
Carribean or Black
Caucasian
Caucasian, Black and Asian
Central American
Chaldean
Chilean American
Colombia
Colombian
Creole French Race
Eastern European
Eg
Egyptian
Eritrean
EspaÃ±ol
Ethiopian
Filipino
Filipino Irish
German, Irish, Filipino, Italian
Greek
Greek, Caucasian native American
Guatemala
Guatemalan
HIspano - Latino
Half ashkenazi half Sephardic jew
Hispana Mexicana
Hispanic
Hispanic/Latina
Human
I don't identify as white!

© 2024 Unger ER et al. *JAMA Network Open*.

In
India
Indian
Indian Subcontinent
Indian origin
Indian/German and Black
Indigenous
Iran
Iranian
Is pano
Jewish
Jewish American
Jewish Dutch Welsh Irish
Jewish Ukrainian
Kenyan American
L
Latin
Latin.
Latin0
Latina
Latinex
Latino
Latino/ Hispanic
Latino/Hispanic/Salvadorian
Latinx
Lebanese
MENA
Mediterranean
Mejicana
Mejico
Mestiza
Mestizo
Mexican
Mexican American
Mexican Indigenous Native
Mexican and middle eastern
Mexican, Russian, Romanian
Mexican-American
Mexican/American
Mexican/Indian/Italian
Mexican/Mayan
Mexicana
Mexicano
MexiffdD1
Middle Eastern
Middle Eastern / Israeli
Middle Eastern, Portuguese
Middle Eastern, Turkish
Middle Eastern/ Egyptian
Middle Eastern/North African
Middle eastern
Middle eastern/Arab
Middle-eastern
Midea
Mix
Mixed

© 2024 Unger ER et al. *JAMA Network Open*.

Mixed (Hispanic and Asian)
Mixed Filipino / Lebanese
Mixed Indigenous, white, and black
Mixed race
Mixed: Indigenous and White
Multi
Multiracial
Multiracial White and Black
Native American
Native American 1/16
Native American/European
Native/Spaniard/African
Negra
North Frican
Norwegian, German, Italian, Native American, Scandinavian
Not american; Croatian
Not defined
Not sure
Not white
Other
Pacific Islander, Asian, white
Persian
Portuguese
Portuguese, Italian, Lebanese
Prefer not to answer
Puerto Rican
South American
South Asian
Southamerican Latino
Spaniard
Spanish
Spanish Origin
Spano
Taiwanese
Trinidadian
Turkic
Turkish
Ugric
Unknown kind
West Indian
West indian
White & Asian
White with substantial Native American heritage
White/West Indies/Native American
White/asian
asian-white mix
black, white, native american
caucacian, greek, native american
central american
half Asian half latin
hispanic
iraqi
italian
japanese/black
latina
latino

© 2024 Unger ER et al. *JAMA Network Open*.

mediterranean
mestiza
middle eastern
mixed
multicultural/multiracial
n/a
other
persian
puerto rican
serbian
spanish american indian /mexican american
spanish latino
syrian middle eastern
unsure, im Peruvian
white/spanish origin

[a] "Other" are free text entered by participants when they didn't find their race in the specified options. Some participants selected other but didn't enter anything. We reviewed the free text to confirm whether free text was duplicated with selections. If the free text was duplicated with selections, then other=0. After review, we only needed to move free text with "Caucasian" to "White" group.

© 2024 Unger ER et al. *JAMA Network Open*.

eTable 2. Observed Distribution of Confounders and Covariates Between COVID-19 Groups

Characteristics	Overall (N=4,738)	COVID+ (N=3,621)	COVID- (N=1,117)	*p*
**Age (at enrollment)**				.04
18 to 34	2,161 (45.6)	1,623 (44.8)	538 (48.2)	
35 to 49	1,647 (34.8)	1,293 (35.7)	354 (31.7)	
50 to 64	930 (19.6)	705 (19.5)	225 (20.1)	
**Gender**				<.001
Female	3,226 (68.1)	2,423 (66.9)	803 (71.9)	
Male	1,437 (30.3)	1,153 (31.8)	284 (25.4)	
Transgender/Non-binary/Other[a]	75 (1.6)	45 (1.2)	30 (2.7)	
**Ethnicity**				.02
No, not of Hispanic, Latino or Spanish origin	4,047 (85.4)	3,117 (86.1)	930 (83.3)	
Yes, of Hispanic, Latino or Spanish origin	691 (14.6)	504 (13.9)	187 (16.7)	
**Race**				<.001
Asian	631 (13.3)	471 (13.0)	160 (14.3)	
Black or African American	513 (10.8)	328 (9.1)	185 (16.6)	
White	3,133 (66.1)	2,464 (68.0)	669 (59.9)	
Other/Multiple[b]	461 (9.7)	358 (9.9)	103 (9.2)	
**Educational Attainment**				<.001
Less than high school diploma	65 (1.4)	43 (1.2)	22 (2.0)	
High school graduate or GED	404 (8.5)	256 (7.1)	148 (13.2)	
Some college but did not complete degree	663 (14.0)	477 (13.2)	186 (16.7)	
2-year college degree	350 (7.4)	251 (6.9)	99 (8.9)	
4-year college degree	1,553 (32.8)	1,258 (34.7)	295 (26.4)	
More than 4-year college degree	1,703 (35.9)	1,336 (36.9)	367 (32.9)	
**Marital Status**				<.001
Never married	1,858 (39.2)	1,346 (37.2)	512 (45.8)	
Married/Living with a part	2,440 (51.5)	1,960 (54.1)	480 (43.0)	
Divorced/Widowed/Separated	440 (9.3)	315 (8.7)	125 (11.2)	
**Family Income (pre-pandemic)**				<.001
Less than $10,000	348 (7.3)	210 (5.8)	138 (12.4)	
$10,000 to $35,000	580 (12.2)	396 (10.9)	184 (16.5)	
$35,000 to less than $50,000	540 (11.4)	382 (10.5)	158 (14.1)	
$50,000 to less than $75,000	658 (13.9)	504 (13.9)	154 (13.8)	
$75,000 or more	2,612 (55.1)	2,129 (58.8)	483 (43.2)	
**Where received COVID test**				<.001
At home testing kit	638 (13.5)	527 (14.6)	111 (9.9)	
Tent/drive-up testing site	2,380 (50.2)	1,984 (54.8)	396 (35.5)	
Clinic including an Urgent Care Clinic	656 (13.8)	449 (12.4)	207 (18.5)	
Hospital	402 (8.5)	288 (8.0)	114 (10.2)	
Emergency department	267 (5.6)	138 (3.8)	129 (11.5)	
Other	395 (8.3)	235 (6.5)	160 (14.3)	
**Tobacco use, past 12 months**				<.001
Daily or near daily	321 (6.8)	217 (6.0)	104 (9.3)	
Weekly	94 (2.0)	67 (1.9)	27 (2.4)	
Monthly	253 (5.3)	201 (5.6)	52 (4.7)	
Less than monthly	81 (1.7)	60 (1.7)	21 (1.9)	
Not at all	3,989 (84.2)	3,076 (84.9)	913 (81.7)	
**Binge drinking, past 12 months**				<.001
Daily or near daily	65 (1.4)	51 (1.4)	14 (1.3)	

© 2024 Unger ER et al. *JAMA Network Open*.

Weekly	487 (10.3)	396 (10.9)	91 (8.1)	
Monthly	1,109 (23.4)	867 (23.9)	242 (21.7)	
Less than monthly	666 (14.1)	533 (14.7)	133 (11.9)	
Not at all	2,411 (50.9)	1,774 (49.0)	637 (57.0)	
**Health Insurance**				<.001
Private & Public	74 (1.6)	57 (1.6)	17 (1.5)	
Private only	3,636 (76.7)	2,867 (79.2)	769 (68.8)	
Public only	835 (17.6)	551 (15.2)	284 (25.4)	
None	193 (4.1)	146 (4.0)	47 (4.2)	
**Hospitalized for Index Illness[c]**				<.001
No	3,478 (73.4)	2,690 (74.3)	788 (70.5)	
Yes	126 (2.7)	118 (3.3)	8 (0.7)	
Missing	1,134 (23.9)	813 (22.5)	321 (28.7)	
**Variant Periods (based on index test date; 50% cutoff)**				<.001
Pre-Delta	818 (17.3)	574 (15.9)	244 (21.8)	
Delta	1,613 (34.0)	1,251 (34.5)	362 (32.4)	
Omicron	2,307 (48.7)	1,796 (49.6)	511 (45.7)	
**Self-reported Comorbidities**				
Overweight or obesity	967 (20.4)	728 (20.1)	239 (21.4)	.35
Asthma (moderate or severe)	466 (9.8)	332 (9.2)	134 (12.0)	.006
Hypertension or high blood pressure	421 (8.9)	311 (8.6)	110 (9.8)	.20
Smoking (currently smoking any type of tobacco, including smokeless tobacco)	169 (3.6)	120 (3.3)	49 (4.4)	.09
Diabetes	168 (3.5)	117 (3.2)	51 (4.6)	.03
Heart conditions, such as coronary artery disease, heart failure or cardiomyopathies	69 (1.5)	48 (1.3)	21 (1.9)	.18
Kidney disease	40 (0.8)	25 (0.7)	15 (1.3)	.04
Liver disease	30 (0.6)	18 (0.5)	12 (1.1)	.03
Emphysema or chronic obstructive pulmonary disease (COPD)	18 (0.4)	9 (0.2)	9 (0.8)	.008
Other conditions in free text[d]	336 (7.1)	246 (6.8)	90 (8.1)	0.15
**Symptoms before index COVID test**				
Fatigue, tiredness, or exhaustion	1,079 (22.8)	673 (18.6)	406 (36.3)	<.001
Problems getting to sleep	1,078 (22.8)	771 (21.3)	307 (27.5)	<.001
Unrefreshing sleep	770 (16.3)	524 (14.5)	246 (22.0)	<.001
Muscle aches/muscle pains	515 (10.9)	331 (9.1)	184 (16.5)	<.001
Pain in joints	351 (7.4)	235 (6.5)	116 (10.4)	<.001
Difficulty thinking or concentrating	293 (6.2)	162 (4.5)	131 (11.7)	<.001
Forgetfulness/memory problems	227 (4.8)	125 (3.5)	102 (9.1)	<.001
Dizziness or fainting	209 (4.4)	127 (3.5)	82 (7.3)	<.001
**New Infections Reported in Follow-up Periods[e]**				
3 Months	196 (5.4)	127 (4.5)	69 (8.7)	<.001
6 Months	213 (6.4)	106 (4.2)	107 (13.9)	<.001
9 Months	212 (8.3)	122 (6.5)	90 (13.8)	<.001
12 Months	242 (13.2)	163 (12.0)	79 (16.7)	.008

[a] Other gender included gender non-conforming, not listed, or prefer not to answer
b Other races listed in free text responses entered by participants are included in the Supplement (eList of Other Races Entered by Participants)
[c] Hospitalization for index illness was a new question added to the 3-month survey after 4/14/2021. There were 1,134 participants missing response to this question that were included as a separate category in the analysis.
[d] Other conditions listed in free text responses entered by participants that could contribute to ME/CFS symptoms. A list of the free text entries is included in the Supplement (eLine List of Conditions Potentially Contributing to ME/CFS Symptoms Entered by Participants).
[e] New infections reported in follow-up periods were not used to calculated propensity scores but adjusted as a time-varying covariate in the GEE model. For 3-12 months, respectively, the total sample sizes were 3613, 3319, 2541, and 1835; the COVID+ group were 2819, 2549, 1888, and 1363; the COVID- group were 794, 770, 653, and 472.

© 2024 Unger ER et al. *JAMA Network Open*.

eFigure 2. Absolute Standardized Differences[a] between COVID-19 Groups (Observed, IPW, Matching)

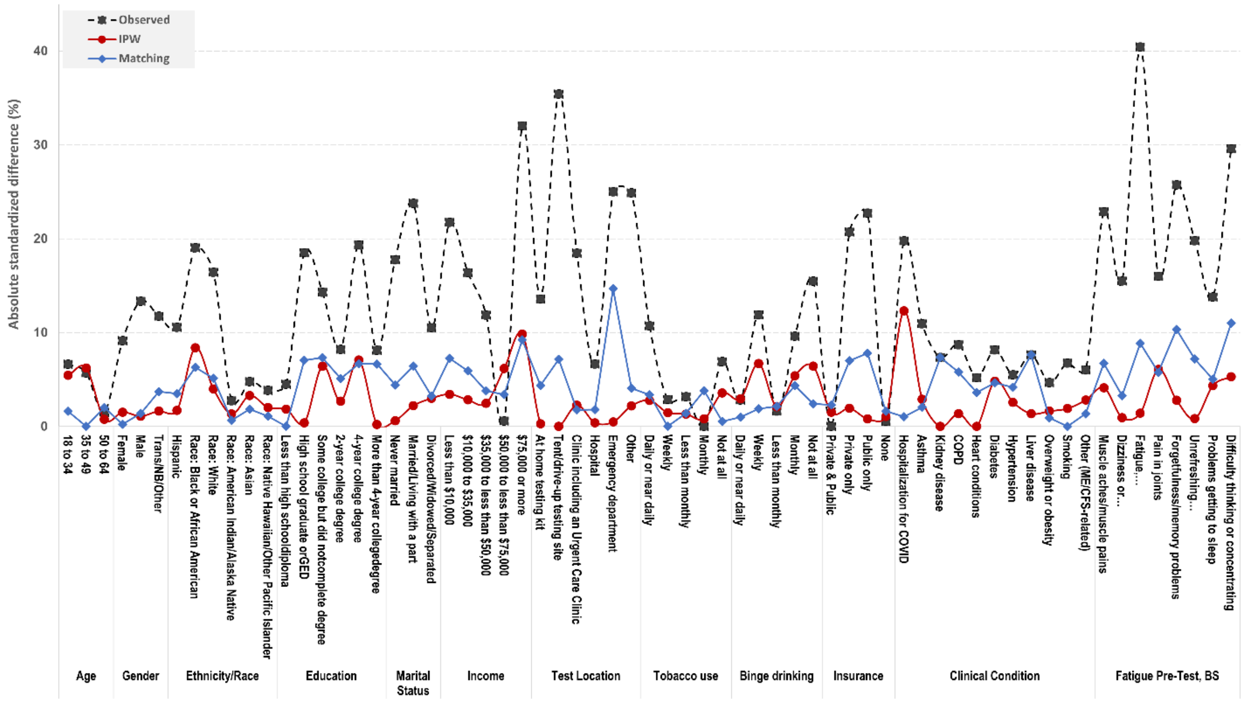

Note: [a] The absolute standardized difference is calculated as $|d| = \left|\frac{(\hat{p}_{covid+} - \hat{p}_{covid-})}{\sqrt{\frac{\hat{p}_{covid+}(1-\hat{p}_{covid+})+\hat{p}_{covid-}(1-\hat{p}_{covid-})}{2}}}\right|$. An absolute standardized difference lower than 10% is commonly regarded as acceptable imbalance.

For both the COVID-positive and COVID-negative groups, the effect size for difference in scores between Ever and Never ME/CFS was moderate to very large for nearly all symptoms at all time points. The effect sizes tended to be larger for the COVID-positive group compared to the COVID-negative group (range 2.72 – 0.1 versus 1.46 – 0.25). For the COVID-positive group, effect size was smallest for dizziness or fainting (0.1 – 0.83) and at some timepoints not significant, and largest for pain interference (2.17 – 2.72) and physical function (2.72 – 2.32). For the COVID-negative group, effect size was smallest for pain in joints at most timepoints (0.27 – 0.5) and largest for physical function at most timepoints (1.46 – 1.30).

© 2024 Unger ER et al. *JAMA Network Open*.

eFigure 3. Marginal Effects of Index COVID-19 Status on the ME/CFS Outcomes Based on Matched[a] Sample

A. ME/CFS-like Illness (0/1)

Comparisons	MOR[b] (95% CI)
FU3: COVID+ vs COVID-	1.12 (0.65-1.93)
FU6: COVID+ vs COVID-	1.06 (0.57-1.94)
FU9: COVID+ vs COVID-	1.42 (0.77-2.62)
FU12: COVID+ vs COVID-	1.10 (0.57-2.10)
COVID+: FU6 vs FU3	0.81 (0.57-1.15)
COVID+: FU9 vs FU3	1.06 (0.73-1.55)
COVID+: FU12 vs FU3	0.91 (0.59-1.40)
COVID-: FU6 vs FU3	0.85 (0.57-1.29)
COVID-: FU9 vs FU3	0.84 (0.55-1.27)
COVID-: FU12 vs FU3	0.93 (0.62-1.39)

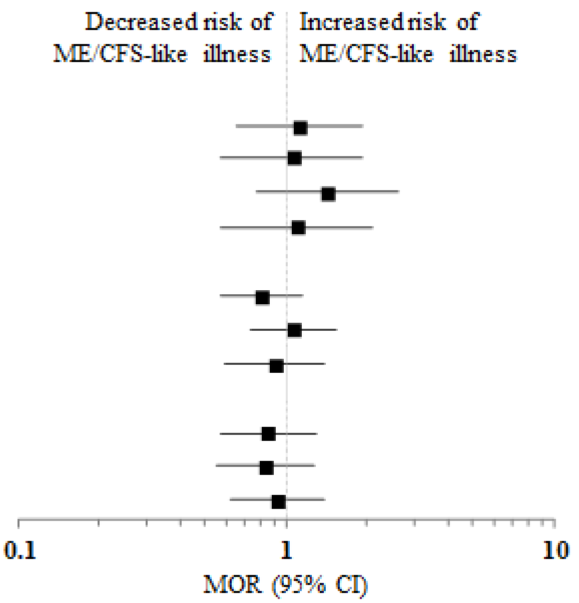

B. Number of ME/CFS Criteria Met (0-5)

Comparisons	MIRR[c] (95% CI)
FU3: COVID+ vs COVID-	1.01 (0.84-1.23)
FU6: COVID+ vs COVID-	0.95 (0.78-1.16)
FU9: COVID+ vs COVID-	0.99 (0.81-1.21)
FU12: COVID+ vs COVID-	0.95 (0.78-1.16)
COVID+: FU6 vs FU3	0.91 (0.82-1.01)
COVID+: FU9 vs FU3	0.98 (0.88-1.09)
COVID+: FU12 vs FU3	0.97 (0.85-1.10)
COVID-: FU6 vs FU3	0.97 (0.87-1.07)
COVID-: FU9 vs FU3	1.00 (0.90-1.12)
COVID-: FU12 vs FU3	1.03 (0.92-1.17)

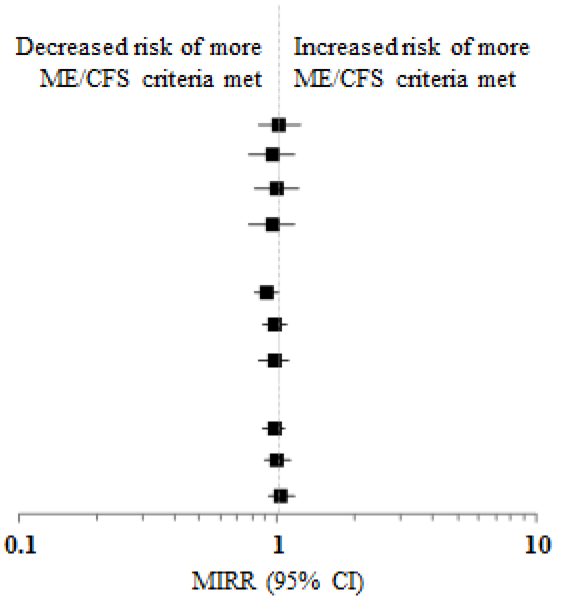

Note: FU3-12 denotes follow-up timepoints at 3, 6, 9, and 12 months after index. Incorporating the technique of inverse propensity score weighting to address the imbalanced characteristics between COVID-positive and COVID-negative groups, the marginal odds ratios and incidence-related ratios were estimated from the general estimating equation (GEE) model that adjusted for initial COVID status (as the exposure variable), variant periods at index COVID test (variant), time points of observations (timepoint), hospitalization status for index illness, subsequent new COVID infections as a time-varying covariate, and the interactions between the exposure variable and the variant, timepoint, and subsequent new infection.

[a] Sample matched on propensity scores between COVID groups

[b] MOR stands for marginal odds ratio

[c] MIRR stands for marginal incidence-rate ratio

© 2024 Unger ER et al. *JAMA Network Open*.
