# Supplementary material for: Myalgic Encephalomyelitis/Chronic Fatigue Syndrome After SARS-CoV-2 Infection
Source: JAMA Netw Open. 2024 Jul 24;7(7):e2423555. doi: 10.1001/jamanetworkopen.2024.23555 (PMC11270135; doi:10.1001/jamanetworkopen.2024.23555)
Supplement: Supplement 3. — Data Sharing Statement [file jamanetwopen-e2423555-s003.pdf]

Data Sharing Statement

Unger. Myalgic Encephalomyelitis/Chronic Fatigue Syndrome After SARS-CoV-2 Infection. *JAMA Netw Open*. Published July 24, 2024. doi:10.1001/jamanetworkopen.2024.23555

Data
Data available: No
